# Categorizing Ideas about Trees: A Tree of Trees

**DOI:** 10.1371/journal.pone.0068814

**Published:** 2013-08-07

**Authors:** Marie Fisler, Guillaume Lecointre

**Affiliations:** URM 7138 CNRS-MNHN-UPMC-IRD “Systématique, Adaptation, Evolution”, Département “Systématique et Evolution”, Muséum National d'Histoire Naturelle, Paris, France; Montreal Botanical Garden, Canada

## Abstract

The aim of this study is to explore whether matrices and MP trees used to produce systematic categories of organisms could be useful to produce categories of ideas in history of science. We study the history of the use of trees in systematics to represent the diversity of life from 1766 to 1991. We apply to those ideas a method inspired from coding homologous parts of organisms. We discretize conceptual parts of ideas, writings and drawings about trees contained in 41 main writings; we detect shared parts among authors and code them into a 91-characters matrix and use a tree representation to show who shares what with whom. In other words, we propose a hierarchical representation of the shared ideas about trees among authors: this produces a “*tree of trees*.” Then, we categorize schools of tree-representations. Classical schools like “cladists” and “pheneticists” are recovered but others are not: “gradists” are separated into two blocks, one of them being called here “grade theoreticians.” We propose new interesting categories like the “buffonian school,” the “metaphoricians,” and those using “strictly genealogical classifications.” We consider that networks are not useful to represent shared ideas at the present step of the study. A cladogram is made for showing who is sharing what with whom, but also heterobathmy and homoplasy of characters. The present cladogram is not modelling processes of transmission of ideas about trees, and here it is mostly used to test for proximity of ideas of the same age and for categorization.

## Introduction

Historians of Science used to categorize schools of thinking without any formal tools to do it (e.g., transformists, evolutionists, gradists, etc.). To produce such categories they had to compare ideas among authors, however, without any clear procedure. The same can be said about systematists, who have been categorizing themselves for long ago into schools like pheneticists, cladists, pattern cladists, synthetists, etc. [1], [2], [3], [4], [5]. Systematics, the science of classification, is using directed acyclic connected graphs to represent relationships among organisms, i.e. hierarchies in the distribution of shared attributes. From those figures systematists produce classifications (nested sets), which categorize organisms. The aim of this study is to explore whether the tools used to produce systematic categories of organisms could be useful to produce categories of ideas in history of science. The benefit should be the reproductiveness and testability of the consistency of the categories that are produced. We propose a testable way to categorize ideas, but we need a case study: what kind of ideas?

We chose the idea of “tree” used across two centuries and half by various authors to represent relationships among organisms. Our basic assumption is that the types of relationships (shown as graphs or branching diagrams) used through time have differed, but they themselves are somehow related. This study therefore focuses on the history of the use of trees to represent the diversity of life and applies to ideas about trees a method inspired from coding homologous parts of organisms. We discretized conceptual parts of the tree; we detected shared parts among authors; we coded those parts into a character matrix and we used a tree representation to show who shares what with whom. In other words, we propose a hierarchical representation of the shared ideas about trees among authors: this produces a “tree of trees” (which is, indeed not the “tree of trees” in the sense of Nye [6]). Then we categorize schools of thinking and discuss a possible phylogenetic interpretation of the representation of shared ideas under the form of a tree.

Authors writing their scientific ideas in books have not only collected data and facts: they also collected information (published or oral) from other authors of different times and places. Scientific ideas can be transferred in a vertical manner from one epoch to another; they can convergently appear at the same time; they can also be “horizontally” transferred in a same epoch from one author to another. When comparing ideas, two questions are then to be considered:

- Are we able to recognize the same idea through different words, and are we able to avoid the pitfall of using the same words used to express different ideas? This is a problem for every historian of ideas, here we propose to formalize shared ideas, i.e. to make explicit through character coding what is often implicitly accepted;
- How to express shared ideas in a synthetic and consistent manner? Some ideas are widely shared; others are restricted to a few authors. A hierarchical distribution of sharings among authors is therefore needed, and a most parsimonious tree seems to be suitable because it shows who is sharing what with whom in a hierarchical manner… but what is the meaning of maximizing consistency among ideas?

Here we promote the formalizing step enabled by the parsimony approach to comparison, in a theory-free framework at least at the beginning (see discussion).

## Materials and Methods

In the scientific field of Natural History we selected 41 books and papers from 1766 to 1991 where the idea of a “tree of life” was expressed and/or illustrated. The period was chosen because the metaphor or picture of “tree” became usual in natural history for those who wanted to organize the diversity of life. These were chosen because they encompass well known and/or fundamental literature in systematics, and they have a theoretical and/or educational content. Some of them even became elements of general scientific culture. Those works may talk about biology, paleontology, or even be philosophical or theological essays. They have in common to present trees of life. The XX^th^ century was not sampled the same way as the XVIII^th^ and XIX^th^. Indeed the explosion of tree reconstruction methods in the second half of the XX^th^ century [7], [8] would require a specific study. Also, we limited the sampling to the modern scientific era. Trees used to support metaphysical views beyond science before the XVII^th^ century (see [9] are out of the scope of the present analysis. In the same way, we chose to limit ourselves to Natural History: we study trees depicting the diversity of life, not trees organizing knowledge (e.g. [10], [11]), languages (historical linguistics) or any other kind of classificatory objects. The 41 works were selected as foreground ones in the field of systematics. The languages selected were English, French and Latin.

### 1. Terminal taxa, or “Operational taxonomic units”

The operational taxonomic units used here are not authors or schools of classification, but books. More precisely, it is what a self-consistent book says or draw about trees. The whole share the property of drawing or describing a classificatory tree based on a theory of classification.

### 2. The Outgroups

We aimed at classifying trees of life. Each of them must be based on a classificatory theory. Linnaeus elaborated a classification of life, but never made trees. For this reason, he was set in the ougroup. But for the same reason, we could not polarize states of characters for the questions about the tree graph. Zaluziansky has drawn dichotomic identification trees, with no objective classificatory purpose. This is the reason why Linnaeus and Zaluziansly have been set as outgroups.

#### 1592: Adam Zaluziansky, “Methodi Herbariae Libri Tres”, (Methods of herbs, manuscript three; only one edition)

Adam Zaluziansky, botanist and doctor, separates botanics from medicine in his *Methodi Herbariae*. In this work, he elaborates several tree-shaped identification keys for the plants he describes.

Zaluziansky has been identified as an outgroup because he created trees, but not taxonomic classifications.

#### 1758: Karl von Linnaeus, “Systema Naturae” (System of Nature), 10th edition

With his *Systema Naturae*, Linnaeus set up a very large classification of life and theorized it. The 10^th^ edition of this work sets up the binomial classification and organizes animals and plants respectively into groups based on anatomical particularities. There is no tree represented in this volume.

### 3. The ingroup

#### 1755: Georges Louis Leclerc, comte de Buffon, “Table des Chiens et de leurs variétés” (Table of the different races of dogs), in. Histoire Naturelle, Générale et Particulière, Tome 5. (Only one edition)

The fifth volume of Buffon's *Histoire Naturelle* describes some domestic animals, including dogs. Following descriptions of dogs, Buffon tries to infer the shape of early, undomesticated dogs. Then, following his idea of “degeneration” and adaptation to climate and natural conditions of species, Buffon elaborates a genealogical table showing dogs migrations and, then, their origin.

This table is to be read neither strictly as a tree nor as a geographic map, but rather as a hybrid of both. A compass is represented on the table, but geographical content does not seem to be respected.

This table is a relative map: it is compass-oriented but does not represent a strict geographic map. Rather, it explains the various transfers that dogs races have undergone. The “chien de berger” (shepherd dog) is considered as the ancestor of all other dogs. The main lines are the first transfers of dogs: to the west, the east, the north, etc. Those transfers produce new dog varieties that are themselves transferred to other countries. Those second lines are much smaller: they do not indicate where dogs have been transferred. As relative coordinates, those lines are to be read as if the card was centered on the firstly-transferred dog, giving to the figure the aspect of an unrooted tree. The table is difficult to understand without Buffon's text, which comes with it.

This table is not a tree. However, Buffon describes it with words and concepts that are commonly used to describe trees, even if the tree is never used as a figure. We can study it as a tree-like extension of maps: it describes a tree-like classification of a part of life, dogs.

#### 1766: Buffon, “De la Dégénération” (About degeneration), in. Histoire Naturelle, Générale et Particulière (Natural History, both general and special), Tome 14. (Only one edition)

Ending the 14^th^ volume of his *Histoire Naturelle*, Buffon describes the phenomenon of “degeneration”, what he explains in terms of mechanisms. There is an “alteration”, as a consequence of the influence of climate, of migrations, etc.; but there is also an internal modification of beings with generations.

Describing this phenomenon, Buffon depicts a tree, grouping together horses, donkeys and zebras. If this figure is never drawn, its description depicts its branches, its root, its ramifications and the mechanism guiding the elaboration of the tree.

This tree is one of the three first occurrences of classificatory trees with a strict methodology, even if it is not drawn but written.

#### 1766: Antoine-Nicolas Duchesne, «Essai sur l'histoire naturelle des fraisiers» (Essay on the natural History of strawberry plants). (Only one edition)

Duchesne is a french botanist from Versailles. Working with his father, he concentrates his studies on strawberry plants, which he cultivates. He experiments with hybridization of strawberries and creates a new variety, which he considers to be a new species.

Duchesne's *Histoire naturelle des fraisiers* firstly investigates the notion of species. Then, he classifies strawberry plants following their supposed genealogy.

In order to fulfill this classification, Duchesne draws a genealogical tree of strawberry plants. He also investigates the idea of strawberry genus and continues with a description of how to culture strawberries.

This tree, one of the three first occurrences of trees of life, is the first drawn one.

#### 1766: Peter Simon Pallas, “Elenchus Zoophytorum” (A review of Zoophytes). (only one edition)

Germano-Russian zoologist, Pallas travelled through Europe. His *Elenchus Zoophytorum*, written in Latin, is a large study of zoophytes. Before this work, Pallas composes a long introduction in the end of which he describes the shape of life classification as being a tree. He strongly argues against the use of scales of beings. A scale of beings is a linear, unidimensional classification of life, generally ending with mankind at its top. The tree he describes is the first global tree of life, grouping plants, animals, and even minerals on its root. The content of trunk and of the main branches is described, as well as the classificatory mode.

Pallas concludes this introduction by strongly recommending the use of such classificatory trees. Interestingly, this written tree has never been drawn by the author.

#### 1770: Buffon, “Histoire Naturelle des Oiseaux” (Natural History of birds), tome 16. (Only one edition)

If the previous occurrence of a tree depiction in Buffon's works was located at the end of the volume, this one is situated in the introduction of the first volume of his *Histoire Naturelle des oiseaux* (*Natural History of Birds*), the sixteenth volume of his *Histoire Naturelle*. The scope of this tree is larger than the previous one: there is no more only a few species that are grouped together, but many avian species that are regarded as being from the same family. Buffon elaborates several trees, each one being the grouping of closely related species. Those family links are as much numerous as the bird species are small.

Buffon links this description to the organization of his work. In this study of birds, he prefers their grouping in genera and their global description versus a description of each species following another.

#### 1774: Johannes Rühling, “Ordines Naturales Plantarum” (Natural orders of plants). (only one edition)

In his *Ordines Naturales Plantarum*, Rühling describes a classification method inspired from other botanists that he then applies to elaborate a succinct classification.

At the end of the preface, Rühling describes and draws a tree indicating “quasi-inbred” affinities between plants. But the classification is not an evolutionary one. Furthermore, the author describes his figure as a geographical table. But this figure is rooted; it contains nodes, branches and leaves. Its shape is the one of a tree.

#### 1790: Johann Wolfgang von Goethe, “Versuch die Metamorphose der Pflanzen zu erklären” (Attempt to explain the metamorphosis of plants). (only one edition)

Better known as a poet, playwright and writer, Goethe has also written a few books and essays about Natural Sciences. In his *Metamorphose der Pflanzen* (*Metamorphosis of Plants*), he studies the affinities between the organs of plants. Those affinities are a metaphor of the affinities and metamorphoses of plants themselves There is a tree-like metaphor of evolution in this work.

#### 1801: Augustin Augier, «Essai d'une nouvelle classification des végétaux» (Experimentation of a new classification of plants). (Only one edition)

French botanist, Augustin Augier elaborates an “*Experimentation of a new classification of plants*”, which is described along with its pitfalls. Augier considers that classificatory tables failed. He then asserts that the only possibility is to classify plants by using a tree that he draws without any evolutionary consideration.

This tree is guided by considerations of values – from the less perfect to the more perfect, in terms of complexity of organization (flowering system, complexity pattern of leaves…), through branches – but also the inclusion and the succession of ranks.

#### 1809: Jean-Baptiste Monet, chevalier de Lamarck, «Philosophie Zoologique» (Zoological Philosophy). (only one edition)

Lamarck's *Philosophie Zoologique* is an epistemological watershed in Life Sciences. In the first part of his work, he describes the mutations of bloodlines along generations of which taxon. Lamarck's theory is the first generalized theory of evolution - even if that word is not yet used in its present meaning. Evolution is seen as a mutation of organs of animals with an increasing complexity of them.

In the text, Lamarck describes his concept that the only possible classification of life is onto a scale of beings. But, in the addenda, he draws a tree illustrating the successive states of bloodlines.

#### 1816: Charles Hélion de Barbançois, “Observations sur la filiation des animaux” (Remarks on the progeny of animals), in Journal de physique, de chimie, d'histoire naturelle et des arts

Barbançois is a follower of Lamarck. In a short paper, he more thoroughly investigates the succession of beings. He organizes them onto his own tree that follows, on the same page, Lamarck's one. Barbançois makes some modifications in Lamarck's classification.

#### 1816: Charles Hélion de Barbançois, “Observations pour servir à une classification des animaux” (Remarks for use in a classification of animals), in Journal de physique, de chimie, d'histoire naturelle et des arts

Barbançois's second article, published a few months after the previous one does not contain the same tree, but much more a hierarchic key which makes gradations in terms of values within each group. He includes humans in his classification (which Lamarck did not do), splitting them into narrow-minded humans and clever ones.

#### 1843: Louis Agassiz, «Recherches sur les poissons fossiles» (Research on fossil fishes), vol. 1. (Only one edition)

Agassiz writes a five-volumes classification of fossil fishes based on the shape of their scales. There, he draws a tree that represents explicitly the occurrences of fossil species through geological times.

Agassiz is not an evolutionist, and he refers to periods of supernatural disappearances and creations of species.

#### 1845: Robert Chambers, “Vestiges of Natural History of Creation”, 3rd edition

Published anonymously, the *Vestiges* work offers a history of Earth and Life. The author proposes a series of theories to explain the world and the universe, including various concepts about the evolution of life. Then, he proposes several recommendations about classification of life. Chambers explains evolution in terms of increasing complexity, and draws a tree to illustrate his words.

#### 1850: Heinrich Georg Bronn, “Recherches sur les lois d'évolution du monde organique pendant la formation de la croute terrestre” (Research on the laws of evolution of the organic world during the formation of the Earth's crust), in Comptes rendus hebdomadaires des séances de l'Académie des sciences. Tome 2

The French Academy of Sciences proposed in 1850 a contest to researchers. They had to elaborate a classificatory system aimed at answering three queries: the position of fossils in sedimentary formations; the question of their appearance and disappearance; and relationships between the present shapes of living forms and the previous ones. The proposed system had to be based on one of the main phyla of life, at least one of the animal classes, but preferably treating life as a whole. The idea was not to propose a theory of evolution, but to offer a classification system suitable for all geological periods.

The price was awarded to Bronn in 1856, which based his system on the observations of successions of beings in strata since Cuvier and D'orbigny's works. The system he elaborates is not an evolutionary one but is based on times of extinctions and creations. Bronn proposes a classificatory tree-shaped system to illustrate his conclusions.

#### 1853: Edward Hitchcock, “Elementary Geology with an Introductory Notice”, 8th edition

Edward Hitchcock was a geologist. In this work, he presents views on geological facts for the public and his students. This book was moreover destined to a Geology congress.

Describing geological data, Hitchcock presents the fossils found in each stratum and then elaborates a classificatory table. This table is then converted into a set of two grouped trees of life, titled “Paleontological chart”. The first one is for animals, the second for plants. Those evolutionary trees are commented with Hitchcock's theory of evolution. In each one, two groups are crowned: Mammalia for animals, with Man on the crown, and Palms for plants.

#### 1855: Alfred Russel Wallace, “On the Law which has Regulated the Introduction of New Species”, in The Annals and Magazine of Natural History

Originally published in the Annals and Magazine of Natural History, Wallace's paper is an attempt to explain the formation of species from other ones. The system given describes the geological, geographical and anatomical arrangement of living forms. The notion of “antitypes”, similar to our “ancestor” notion, guides the theoretical principle.

To illustrate those ideas, Wallace describes a theoretical figure, openly analogous to a branching tree, which is not drawn.

#### 1856: Alfred Russel Wallace, “Attempts at a Natural arrangement of Birds”, in The Annals and Magazine of Natural History

After a study of birds in Southern America, and during another one in the Indian Islands, Wallace begins a classification of bird groups. The finished work is not an evolutionary one but rather an arrangement of groups according to their morphological affinities.

In this article, Wallace draws two classificatory unrooted trees of birds.

#### 1859: Charles Darwin, “On the Origin of Species”, 1st edition

In this founder work for modern biology, Darwin proposes a theory for the evolution of species and their classification. The famous tree from that book functions as a conjecture about the general shape of the genealogical links if the theory is true [22], and it is followed by the required consequences for classification. Its status is theoretical. The first edition of this work is studied separately from the last one and appears in separate rows in the data matrix, because of numerous modifications that have been done in between. Among these changes, Darwin clearly expressed a requirement for monophyly of groups in the first edition [13] [14], a section that was removed in the sixth edition.

#### 1866: Albert Gaudry, «Considérations générales sur les animaux fossiles de Pikermi» (General considerations on the fossil animals of Pikermi). (only one edition)

Albert Gaudry was a paleontology professor at the French National Museum for Natural History, which he directed. In 1866, he published a genealogical classification of Pikermi (Greece) fauna. Benefiting from exceptionally preserved fossils, he attempted to arrange them following Darwin's prescriptions. His tree, considered as the first use of Darwin's one used for classifying, was lauded by Darwin.

Gaudry did not consider natural selection to be true, but rather believed in a deistic-guided harmony and regulation.

#### 1866: Ernst Haeckel, “Monophyletischer Stammbaum der Organismen”, in Generelle Morphologie der Organismen, (General morphology of organisms). (only one edition)

In this early work, Haeckel draws the famous monophyletic tree of life that is divided into three kingdoms following the three domains of study of life: Plants, Animals and Protists.

#### 1868: Ernst Haeckel, “Natürliche Schöpfungsgeschichte” (The History of Creation), translation of the 6th edition

In his *History of Creation*, Haeckel studies the history of the theories of evolution and proposes an inventory and a review of modern knowledge in biology. There, he develops his theory of ontogeny, which he links to the phylogeny. Then, defining the term of phylogeny (the first edition of this work was published in 1868, before Anthropogenie), he performs a phylogenetic classification of life with numerous genealogical trees. Finally, he deals with the place of mankind among living organisms and a racialist classification of humans.

#### 1874: Ernst Haeckel, “Anthropogenie”, 1st edition

*Anthropogenie* is a history of the development of mankind. Haeckel uses the idea of ontogeny, explained by phylogeny, to trace the history of the human bloodline. Organ after organ, he achieves a history of the improvement of the main body parts into evolutionary steps up to the development of mankind.

He illustrates his work by a genealogical tree of mankind, divided into a succession of genealogical groups.

#### 1882: Anton Reichenow, “Vögel der zoologischen Gärten” (Birds of zoological gardens). (only one edition)

This German volume was intended for bird-breeders. It presents a treelike classification of extant birds.

#### 1876: Charles Darwin, “On the Origin of Species”, 6th edition

Even if this posthumous last edition of *The Origin of Speci*es is very similar to the first one in its content, many corrections and rewritings have been done since. If the figure of Darwin's tree is exactly the same, the epistemological content of the text has been slightly modified. This is the reason why the first and the last edition of Darwin's Origin of Species have been studied as two different operational taxonomic units.

#### 1940: Lucien Cuénot, “Un essai d'arbre généalogique du règne animal” (An attempt of genealogical tree of animals), in Revue Scientifique

Lucien Cuénot describes a genealogical tree of animals. Aimed at illustrate a classification of life, this colored figure had a high educational purpose: it was even presented in a French science Museum, the *Palais de la Découverte*.

In this article, Cuénot describes the ideas directing the elaboration of the tree. He lends his own considerations about a direction in evolution and the idea that it is finished: there is no more evolution.

#### 1953: Ernst Mayr, “Methods and Principles of Systematic Zoology”. (Only one edition)

Ernst Mayr wrote a treatise about the principles of numerical taxonomy. This one is destined to teachers, biologists, and also amateurs. After a brief history of taxonomy, Mayr elaborates taxonomic procedures from the collection of specimens to the elaboration of taxonomic papers. He describes here the different analysis methods, and includes several trees in order to describe their elaboration. Then, he describes the process of zoological nomenclature following international rules.

#### 1955: Pierre Teilhard de Chardin, «Le Phénomène Humain». (The human phenomenon). (only one edition)

Teilhard de Chardin, paleontologist and Theologian, wrote two books aimed at explaining the conciliation between scientific knowledge and his personal religious believes. In this first one, written in 1947 and published as posthumous, he writes a history of the Universe. Linking theories in Physics and Biology with personal convictions, he writes an “introduction to an explanation of the world” and an attempt of general explanation of evolution. Teilhard de Chardin elaborates a theory to accommodate Darwinian natural selection to degrees of higher encephalization given to some species, until a cosmic «omega” point. He represents this theory with several tree drawings.

#### 1956: Pierre Teilhard de Chardin, “Le Groupe Zoologique Humain” (the human zoological group). (only one edition)

In this second work, Teilhard de Chardin concentrates his study on mankind as a “phenomenon”. He attempts at assigning the place of *Homo sapiens* in nature, among other forms of life. Teilhard de Chardin writes a story of the anthropogenesis in five steps: life in the universe, biosphere, appearance of mankind, expansion step of his “noosphère” and then its compression. More than a study of the past, the author aims at giving an interpretation of the appearance of mankind, and its future in a theological interpretation of life.

#### 1962: George Gaylor Simpson, “Principles of animal taxonomy”. (2nd impression)

In this work, Simpson develops classificatory models. From the original data to the tree and the classification, he gives instructions to proceed. This book is less an essay on classifications than a kind of handbook for students or researchers. There are several kinds of trees illustrating each step of the elaboration of a taxonomic work.

#### 1963: Robert Sokal & Peter Henry Andrews Sneath, “Principles of Numerical Taxonomy”, 1st edition

How can taxonomists make non-arbitrary groups? Tough they are convinced by evolution, Sokal and Sneath claim the impossibility to find the phylogeny of species. But they advocate for tree-construction methods based on global similarity. Characters are not directly treated as such onto the tree because they have been previously mixed into a pairwise distances matrix. Thus their “phenograms” aren't phylogenies, what is assumed by the authors, but one of the first attempts to mathematize and objectivize the elaboration of taxonomic groups.

#### 1966: Alfred Romer, “Vertebrate Paleontology”, 3rd edition

This work condenses numerous data about vertebrates. Including many paleontological data, it proposes tree-shaped classifications of vertebrates.

#### 1966: Robert Sokal, “Numerical Taxonomy”, in Scientific American

Sokal's article develops the first principles of numerical taxonomy to match to new computation possibilities given by computers. Sokal develops much more this theory to make it able to classify imaginary animals, the famous “Caminalcules”. Then, he proposes a mathematically and similarity-based ranking method.

#### 1966: Willi Hennig, “Phylogenetic Systematics”. (Only one edition)

This English version of Hennig's “*Grundzüge einer Theorie der phylogenetischen Systematik*” proposes a novel classification method. It aims at reconstructing phylogeny, given as knowable, without the need of inclusion of pre-conceived groups into the procedure. He redefines monophyly, paraphyly and polyphyly and concepts of species and higher taxonomic groups.

Hennig's Phylogenetic Systematics has become the basis of modern classifications.

#### 1967: Alfred Romer, “Major Steps in Vertebrate Evolution”, in Science

Romer explores, in this article, the origin of modern man in a succession of “major steps” since a “metazoan ancestor”. He aims at reconstructing this sequence of selective steps from tunicates to vertebrates and from the rise of a bony structure to the emergence of terrestrial higher vertebrates, and then to primates and mankind.

#### 1973: Alfred Romer, “l'origine des classes de vertébrés” (“The origin of Vertebrate classes”), in La Recherche

Are amphibians descended from a single common ancestor, or are they a polyphyletic group? In this article, Romer discusses, in terms of emergence of groups from other ones, the development of vertebrates and the search of intermediate forms. From fossils and paleontological data, he elaborates two trees: the first one for vertebrates, the second for tetrapods. Then, he discusses the production of “natural classes”, descending from a common ancestor.

#### 1973: Sokal & Sneath, “Numerical Taxonomy”, 2nd edition

Ten years have passed since Sokal and Sneath's first Numerical Taxonomy. Technology has lead to the appearance of computers in laboratories, enhancing computational possibilities. Epistemological and mathematical novelties have been developed, such as the parsimony algorithm. Above all, Hennig's Phylogenetic Systematics emerged in the English-speaking world and totally remodeled the methodological landscape of Systematics.

The authors have corrected and enriched their own considerations. Especially, they develop in this work the idea of “numerical cladism” and its methodologies.

#### 1982: Ernst Mayr, “The Growth of biological thought: diversity, evolution and inheritance”. (Only one edition)

Almost twenty years after the rise of cladism, after ten years of intense debates among the classificatory schools of pheneticists, cladists and synthetists, time has come to write a new history of biology. Mayr writes three books to do so. This one is about Evolution.

Well-known for his gradist classifications, Mayr casts a critical eye on other schools. This epistemological work deals about clades, grades, and their representation onto trees.

#### 1988: Eliott Sober, “Reconstructing the Past”. (Only one edition)

Reconstructing the Past summarizes the most recent methodological discoveries since the Hennigian revolution. From a philosophical and epistemological point of view, it discusses the implementation of the parsimony principle and its achievements. Moreover, it explores the suitability of statistical methodologies for cladistics.

#### 1991: Pascal Tassy, “L'arbre à remonter le temps” (The tree that goes back in time), 1st edition

Tassy, paleontologist, writes a popularization work about the history of trees of life and their contemporaneous use and elaboration. His critical views about his predecessors enable us to code his own ideas about trees into our matrix.

The corresponding list of analyzed sources is therefore the following:

Agassiz L (1843) Recherches sur les Poissons Fossiles, tome I. Neuchâtel: Imprimerie de Petitpierre.

Augier A (1801) Essai d'une nouvelle classification des végétaux conforme à l'ordre que la nature parait avoir suivi dans le règne végétal. Lyon: Bruyset aîné et comp.

Barbançois CH (1816) Observations sur la filiation des animaux depuis le polype jusqu'au singe. Journal de physique, de chimie, d'histoire naturelle et des arts 82: 444–448.

Barbançois CH (1816) Observations pour servir à une classification des animaux. Journal de physique, de chimie, d'histoire naturelle et des arts 83: 67–78.

Bronn HG (1861) Essai d'une réponse à la question de prix proposée en 1850 par l'Académie des sciences pour le concours de 1853, et puis remise pour celui de 1856. Comptes rendus hebdomadaires des séances de l'Académie des Sciences, suppl.2: 377–918.

Buffon G (1755) Table des chiens et de leurs variétés. In: G. Buffon (Ed.) Histoire naturelle, générale et particulière, tome V. Paris: Imprimerie Royale. pp. 311.

Buffon G (1766) De la dégénération. In: G. Buffon (Ed.) Histoire naturelle, générale et particulière, tome XIV. Paris: Imprimerie Royale, 311–374.

Buffon G (1770) Histoire naturelle des oiseaux, tome XVI. Paris: Imprimerie Royale.

Chambers R (1844) Vestiges of the natural history of creation and other evolutionary writings. 1st ed. Reprinted (1994). In: J. Secord (Ed.) Chicago: University of Chicago Press.

Cuénot L (1940) Un essai d'arbre généalogique du règne animal. La Revue Scientifique 78: 223–229.

Darwin C (1859) On the origin of species by means of natural selection. John Murray, London. First Edition. Penguin Classics, 1985, London: Penguin Books.

Darwin C (1876) On the origin of species by means of natural selection. London: John Murray.

Duchesne N (1866) Histoire naturelle des fraisiers. Paris: Didot le Jeune et C.J. Panckoucke.

Gaudry A (1866) Considérations générales sur les animaux fossiles de Pikermi. Paris: F. Savy.

Goethe JW (1790) Essai sur la métamorphose des plantes. Traduit de l'allemand sur l'édition originale de Versuch die Metamorphose der Pflanzen zu erklären, Gotha par M. Frédéric de Gingins-Lassaraz, 1829. Genève: Barbezat.

Haeckel E (1874) Anthropogénie. Traduit de l'allemand sur la 2e édition de l' Anthropogenie par le Dr Ch. Letourneau, 1877. Paris: C. Reinwald.

Haeckel E (1866) Generelle morphologie. Berlin: G. Reimer.

Haeckel E (1868) Histoire de la création des êtres organisés, d'après les lois naturelles. Traduit de l'allemand sur la seconde édition du Natürlichen Schöpfungsgeschichte par le Dr CH. Letourneau, 1877. Paris: C. Reinwald.

Hennig W (1950) Grundzüge einer Theorie der phylogenetischen Systematik. Berlin: Deutscher Zentralverlag.

Hennig W (1966) Phylogenetic Systematics. Urbana: Univ. of Illinois Press.

Hitchcock E (1853) Elementary Geology with an introductory Notice, 8^th^ edition. New York: Newman and Ivison.

Lamarck JB (1809) Philosophie Zoologique. Paris: Dentu.

Linnaeus C (1758) Systema naturae. Editio Decima. Impensis Direct. Stockolm: Laurentii Salvii.

Mayr E (1982) The Growth of biological thought: diversity, evolution and inheritance. Cambridge: Harvard University Press.

Mayr E (1953) Methods and principles of systematic zoology. New York: McGraw-Hill.

Pallas PS (1766) Elenchus Zoophytorum. Den Haag: Apud Petrum van Cleef.

Reichenow A (1882) Die Vögel der zoologischen Gärten. Leipzig: L.A. Kittler.

Romer AS (1966) Vertebrate paleontology. Chicago: University of Chicago Press.

Romer AS (1967) Major steps in vertebrate evolution. Science 158: 1629–1637.

Romer AS (1973) L'origine des classes de vertébrés. La Recherche 33: 347–361.

Rühling J (1774) Ordines naturales plantarum commentatio botanica. Goettingen: Sumtibus vid. Abrah. Vandenhoeck.

Simpson GG (1962) Principles of animal taxonomy. New York: Columbia university press.

Sober E (1988) Reconstructing the past. Massachusets Institute of Technology Press. Cambridge.

Sokal RR (1966) Numerical Taxonomy. Scientific American 215(12): 106–116.

Sokal RR, Sneath PHA (1963) Principles of numerical taxonomy. San Francisco: W.H. Freeman.

Sokal RR, Sneath PHA (1973) Numerical Taxonomy. San Francisco: Freeman. Anderberg, M. R.

Tassy P (1991) L'arbre à remonter le temps. Paris: Christian Bourgois.

Teilhard de Chardin P (1956) Le groupe zoologique humain. Paris: Albin Michel.

Teilhard de Chardin P (1955) Le phénomène humain. Paris: Éditions du Seuil.

Wallace AR (1856) Attempts at a Natural Arrangement of Birds. The Annals and Magazine of Natural History, second series 105: 193–214.

Wallace AR (1855) On the Law which has Regulated the Introduction of New Species. Annals and Magazine of Natural History, second series 16: 184–196.

Zaluziansky A (1592) Methodi herbariae Libri Tres. Francofurti: E Collegio Paltheniano.

### 4. Vocabulary used

The analysis is based on 91 characters. Characters descriptions involve some specific vocabulary, which is explained here.

#### Blob, Bubble

A blob, or a bubble, is a bidimensional baloon. A “bubble tree”, or “romerogram”, is a tree made of such kinds of elementary objects, either linked to one another (e.g. in Romer) or independent (e.g. in Agassiz). Rather than linear branches, “bubble trees” are successions of closed two-dimensionnal shapes.

#### Discontinuities

A discontinuity is a gap, lack of continuity between two elements. Those elements can be genealogical links, blobs, groups based on global similarity, or in a chain of beings… Discontinuities are not “vertical cuts” (below): cuts are lines, not gaps.

#### Diversification axis

This axis of a tree represents diversification, i.e. the number of independent lines to the terminal leaves. This number, along this axis, does not increase with hierarchical resolution. An unresolved tree of 7 terminals has a value of 0 in the hierarchical axis and a value of 7 in the diversification axis. A fully resolved tree of 7 terminals has a value of 5 in the hierarchical axis and always a value of 7 in the diversification axis.

#### Groups

Groups are basically sets of objects gathered according to a given consistency. Thereby, for example, for phylogenetic systematics, taxa are sets of individuals grouped according to a principle of monophyly.

#### Hierarchical axis

The hierarchical axis of a tree is the one that counts the number of levels of inclusion implied by the various groups (or ranks), i.e. by the hierarchical resolution. An unresolved tree of 7 terminals has a value of 0 in the hierarchical axis and a value of 7 in the diversification axis. A fully resolved tree of 7 terminals has a value of 5 in the hierarchical axis and a value of 7 in the diversification axis.

#### Leaf objects (or “tips”)

The objects set on leaves are the kind of elementary entities classified. These can be taxa (when dealing with life), musical instruments, ideas… If they are taxa, they can be of several kinds: classes, families, species, populations, or openly one specimen.

#### Properties

Properties are attributes, qualities or characteristics of the classified objects.

Steps. A step is an evolutionary stage. It generally implies a progression toward a goal, a more complex evolutionary stage.

#### Value

An “evolutionary value” is the quality of something that renders it, *in fine*, closer to mankind's abilities.

#### Vertical cut

A “vertical cut” is a vertical *line* that cuts a tree into «paraphyletic” groups, i.e. a node but not all its descending branches. A vertical cut is not what we call above a “discontinuity”. A discontinuity is a gap, not a line.

### *5.* Characters classified into thematic areas

To allow a better understanding of the ideas that are coded into the character matrix, we classified the 91 characters into five thematic areas:

The elements of the tree,

The meaning of the tree,

The content of the tree,

Trees and taxonomy,

The methodology employed.

Through these five thematic areas, and to facilitate understanding of characters, the characters are themselves grouped into 33 sections. Each of them is a question about the tree.

#### Elements of the Tree

Here we detail each component of the tree, whether they are general (root, leaves, branches, nodes, etc.) or specific to certain authors (“blobs”, cuts across the tree, etc.). We also code the meaning of the tree root in terms of whether it implies an object or concept. If its meaning is an object, it is either considered as being (or having been) alive or an inert entity (such as minerals, molecules, etc). If the root refers to a concept, it is either an initial state of characters or a type.

Section “Meaning of the root” (Characters 1 to 7). *The root is the base of the tree. It is the point from which emerge the first branches and thus all the potential extent of their diversification*.

Section “Meaning of the lines” (i.e. branches; Characters 8 to 10). The term “lines” refers to the branches of trees. They represent links between roots, leaves or trunk. Links carry a message: they are the expression of a purely logical relationship among objects or branches bear a content. This content changes along the branch: bloodlines, or gradation (or not) in terms of perfection.

Section “Meaning of the internal nodes” (Characters 11 to 14). Nodes are, topologically, the point from where branches emerge. As they are a-dimensional objects, they cannot express any gradation. However, they carry information. As trees express relationships among the various entities of life, nodes can refer to species, races and varieties or groups of larger size. They can also bear concepts: mass extinctions, characters, etc. Finally, nodes are sometimes linked to a notion of ancestor: these two ideas can then be confused and a “concrete” ancestor be placed at this point.

Section “Meaning of the leaves” (Characters 15 to 19). Leaves are, in a tree, objects connected to a line and only one. They are moreover located opposite to the root. In addition, leaves can be related either to current times or past ones. They can explain the end of an evolutionary pathway, its finality or merely its most recent expression. Indeed they can furthermore be the expression of an evolutionary destiny, a teleological apex of evolution, the ultimate ending of a progression. Leaves may symbolize groups or species. They can also consider time, reflecting changes in species and their reasons. Finally, some of authors regard leaves as the only detectable objects.

Section “Meaning of vertical cuts” (Character 20). A “vertical cut” is a split between two parts of the tree. If present, they symbolize a break-up between either groups or kingdoms.

Section “Meaning of “blobs” (Characters 21 to 22). A “blob”, or a “bubble”, is a swelling in size into the tree. They most often replace branches. As bi-dimensional objects – branches are uni-dimensional ones – they so express a second message. Bubbles may thus refer to a numerical quantity of species within a given group over the generations. They may furthermore bear a message of gradation.

Section “Meaning of the ancestor” (Characters 23 to 26). With the idea of a chronology in evolution comes the one of the ancestor. This one is narrowly linked to a concept of descent.

**Figure 1:**
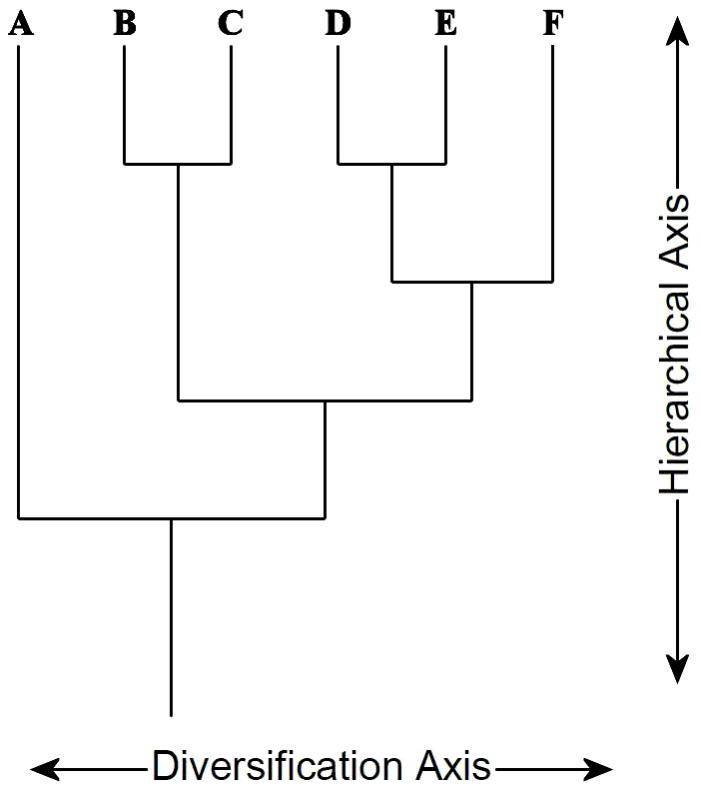
Virtual tree showing what we call the “Diversification axis” and the “Hierarchical axis”. Both axes increase with the number of terminals when the tree is fully resolved. When it is not, the diversification axis increases while the hierarchical axis does not.

Section “Orientation of the tree” (Characters 27 to 31). How is the tree to be read? There are rules guiding the reading of the tree, and those one admit an orientation for reading – basically from the root to the leaves. However, a tree is always drawn in two dimensions and we need here to name the two dimensions. The dimension along which hierarchical levels are embedded into one another can be called the “hierarchical axis” (Fig. 1). This axis increases in number of steps (or in length) as the hierarchical resolution increases. This is, most often, the axis along which authors place time when it is the case. The other dimension is called here “diversification” axis. This axis does not increase in number of steps (or length) when the hierarchical resolution increases. It increases only with the number of leaves.

Section “Diversification axis of the tree” (Characters 32 to 35). There is a message set on the two axes of the tree, its abscissa and its ordinate. The diversification axis may take various meanings. It may thus symbolize just diversification of species or groups from their common base, but may also illustrate properties or a value gradation in a value system. It may exceptionally be the result of the necessity of nesting different groups into each other (in Haeckel or Wallace). Finally, on the diversification axis may also contain a notion of time (for example in Haeckel).

Section “Hierarchical axis of the tree” (Characters 36 to 40). This character is independent from the characters 28–32. Indeed the meaning given to the diversification axis is independent from the meaning given to the hierarchical axis.

Section “Well-marked discontinuities” (Characters 41 to 42). Drawing a tree implies accepting a continuity and a discontinuity in life. A “well-marked” discontinuity must be identified by an empty space between two branches of the tree. Discontinuities are not splits superimposed to the graph; they are empty spaces into the graph itself. They may appear either horizontally or vertically.

#### Meaning of the tree

What is the tree made for? We will focus on the use of trees for classification.

Section “Classificatory aim” (Characters 43 to 44). Has the tree been drawn in order to classify a content? (in opposite to a merely illustrative tree, for example).

Section “Status of the tree's graph” (Characters 45 to 46). As mentioned above, trees can have a theoretical and/or an epistemological status: epistemological trees propose classifications of concrete objects, animals, plants, sharks, etc. On the opposite, a theoretical tree models the shape that a classification might have if the theory about the processes of diversification is true. Trees can be only theoretical, only epistemological, or be a combination of the both.

Section “Does the tree intends to illustrate the natural order?” (Characters 47 to 48). An intrinsic “order of the Nature” is supposed and is what classification is expected to reflect using a tree. Moreover this term “natural order” must be explicitly mentioned. Note that the link between the order in Nature and classificatory purposes is not unequivocal. For instance, for pheneticists there is a natural (genealogical) order in Nature however it is not what classification intends to reflect.

Section “The tree is genealogical” (Characters 49 to 50). The tree is theoretically based on a genealogical background: if species do evolve, there thus must be bloodlines, implying kinship. We must define genealogy here. It is a ancestor–descendant relationship between two concrete individuals. However here that relationship can be either thought as merely theoretical or thought by the author as a concrete empirically accessible link.

Section “Are Beings steps of a value system?” (Characters 51 to 52). In some authors there can be a value gradation among beings reflecting a value system; for instance gradation in perfection. The notion of perfection is then expressed through various means: roots, branches, bubbles, leaves, clippings, ordinate or abscissa.

Section “Teleology: direction in evolution?” (Characters 53 to 54). Is evolution guided by a specific direction or is it the mere product of fortuity?

#### Content of the tree

We will focus here on contents of a tree: fossils, kingdoms, ancestors, etc. That's what is carried – or not – by the tree which will target our interest.

Section “Do fossils belong to the classification?” (Characters 55 to 56). Are fossils taken into consideration, or merely ignored?

Section “Trees take into consideration the extinctions of species” (Character 57). Do trees consider the possibility of the complete disappearance of a species?

Section “Is the ancestor concrete?” (Characters 58 to 59). A “concrete” ancestor is an organism, extinct or alive, assigned as the ancestor of a group. Its body, complete or lacunar has been found and a name has been attributed to it.

Section “Consideration of time in the tree” (Characters 60 to 61). Larger than the mere genealogy, is there a notion of time carried by the tree? Here we code 1 if time is present whatever the axis (hierarchical or diversification, see characters 36 and 39).

Section “Kinship between plants and animals?” (Characters 62 to 63). One of the most recurrent questions in ancient authors is the relationship between plants and animals. It is therefore necessary to examine relationships between the two life's kingdoms that are embedded into our pre-scientific cultural background.

Section “Ability to interbreed” (Character 64). In some ancient authors interbreeding is the ability for groups (larger than mere species) to cross one another. A global ability to interbreed is never found. Meanwhile, the question is here to know if interbreeding (including between distant groups) is possible or not.

Section “Extent of the tree” (Characters 65 to 66). The tree aims here at studying a larger or a smaller group. Is the group studied the “largest” one, including all beings, or does the tree incarnate only a small part of life? Typically, some authors make a distinction between plants and animals in the applicability of their method, which restrict the extent of the classificatory program. In some works, there are several trees; one for each different part of life.

Section “Special position assigned to mankind” (Characters 67 to 68). Is mankind set at the top of the tree? Is its place a privileged one? This question is recurrent among authors.

#### Trees and taxonomy

Obtaining data and relationships is not an isolated activity. Authors may also create groups.

Section “Properties of groups” (Characters 69 to 78). How are groups composed? What are they made of?

Section “What do classificatory ranks express?” (Characters 79 to 80). How are classificatory ranks and taxonomic “arrangements” related?

Section “Reality given to categories” (Characters 81 to 82). Are the categories given as real, non-arbitrary elements of life, or are they seen as arbitrary concepts? (for instance, categories are real for Linnaeus and closer to us, for Dubois [43] the genus is a real evolutionary unit in Nature).

#### Methodology Employed

Section “Use of parsimony (character 83). Although this criterion is, from all, the most recent one, the use of parsimony principle appears as being rich enough to be set in our characters matrix. The use of this principle must be openly asserted.

Section “Classification based on presence of characters” (Characters 84 to 85). Is classification based on presence or lack of characters?

Section “Classification (i.e. arrangement) by global similarity” (Characters 86 to 87). A classification can also be elaborated according to global similarity.

Section “Use of monophyly” (Characters 88 to 89). Monophyly is the property of grouping to an ancestor all its descent and nothing less; to group entities by the mere consideration of bloodlines. If the modern meaning of this term is rather recent, we will here consider the idea of monophyly, whatever its name could be.

Section “Consideration of homoplasies” (Characters 90 to 91). Homoplasies are similar character states that have not been inherited from a common ancestor.

### 6. Description of characters

#### Character 1: A concrete species or ancestral group is referred to at the root

A concrete form is seen as a being able to reproduce its own kind. Here, this is a concrete being that the author assigns to the root. This is, for example, what Darwin does (0), whereas Hennig does not.

Yes = 0; No = 1

#### Character 2: Initial states of characters at the root

Initial states of characters are a list of components supposed to characterize the object carried by the root. Here, we don't have a concrete being at the root but only a list of some of its properties. On the opposite from the previous character, Darwin does not do so, whereas Hennig does.

No = 0; Yes = 1

#### Character 3: Inorganic form (minerals, …) at the root

An inorganic form, as minerals, is unable to reproduce its own kind. Even if, historically, minerals have sometimes been seen as parts of life, we have distinguished minerals from living beings. Thus, Bronn and Hitchcock see inorganic forms at the “root” of the tree.

No = 0; Yes = 1

#### Character 4: If the root carries the idea of a being: is this one still alive (even as a “living fossil”) or extinct?

As an example, Buffon considers that the being set at the basis of the tree is still alive (the horse, the shepherd dog…) whereas, according to Darwin, this species is extinct.

Extant = 0; Extinct = 1

#### Character 5: Root taxon of higher value

A “value” is ideological expression. A value is given according to a value system, most often implicitly. Here a value is some combination of the ideas of perfection and complexity. In the case where the root starts a value gradation, the other entities are then viewed as “degenerate”, or less perfect than the original entity. Is the taxon at the root seen as more complex and/or more perfect than the others on the tree? Buffon adheres to this idea, whereas Darwin or Lamarck disagrees with it.

No = 0; Yes = 1

#### Character 6: Root taxon of lesser value

In the case where the root starts a value gradation, the other entities are then viewed as more progressive and/or complex than the original entity. Is the taxon at the root seen as less complex and/or less perfect than the others on the tree? Lamarck thinks so whereas Darwin does not.

No = 0; Yes = 1

#### Character 7: No value

There is absolutely no gradation in terms of value between the root and the rest of the tree. Darwin adheres to this idea, whereas Chambers disagrees with it.

Yes = 0; No = 1

#### Character 8: Genealogical kinship links

The link between the two extremities of the line is conveyed by a genealogical process. There is an idea of descent that is set into branches. For instance this idea is not accepted by Pallas or Augier, but it is by Darwin or Haeckel.

No = 0; Yes = 1

#### Character 9: Purely logical links

A “logical link” is the use of branches in order to represent only a hierarchy. Hierarchies in the form of a tree are used to express the sharing of features, identification keys, etc. Hennig or Agassiz use such links, whereas Buffon or Darwin do not.

Yes = 0; No = 1

#### Character 10: Value gradation within a branch

Are branches tools to express a value system? In other words, do branches express differences in values among entities? If yes, there are differences between the values of the entities at the beginning and at the end of the branch. Note that trees that depict an overall gradation in value at the scale of the whole tree (character 51) do not necessarily include such a gradation within a single branch: character 10 is not redundant with character 51. As an example, Buffon considers that there is such a gradation whereas Darwin does not see any gradation within branches.

No = 0; Yes = 1

#### Character 11: Nodes as species, races, varieties

Do internal nodes refer to populations or individuals that are able to interbreed? This is the case with Hennig but not with Haeckel, for example.

No = 0; Yes = 1

#### Character 12: Nodes as groups of higher rank

Nodes are groups above the mere species level. It can be genera, or higher-ranked groups. If the rank isn't mentioned, a “group” is essentially composed of several species. Such groups at nodes give birth to other groups without including them (they are not groups in a cladistic sense). Lamarck or Hitchcock see such groups at nodes, whereas Hennig or Darwin do not.

Yes = 0; No = 1

#### Character 13: Nodes as concepts

Nodes are “concepts”, either a list of characters or a property. A “concept” may be a hypothetical common ancestor, a reconstructed entity, character states, or a property used in a determination key. Note that the informative content of a node in a phenogram could appear difficult to interpret at the first glance. Clearly the node of a phenogram is made of global similarity, and as such it is a concept. This idea is not accepted by Haeckel, but it is by Sokal and Sneath.

No = 0; Yes = 1

#### Character 14: When the node corresponds to an ancestor, is it actually found or not?

Has the “ancestor” been found? Is it a discovered fossil? Gaudry thinks so whereas Darwin does not.

No = 0; Yes = 1

#### Character 15: Group at the leaves

A group at the leaves has an upper rank than the mere species. If the rank isn't specified, a group is basically composed of several species. Darwin considers that species are at the leaves whereas Buffon sees groups on leaves.

Groups = 0; Species = 1

#### Character 16: Time taken into consideration

Are leaves set into a time framework? Are they inserted into the chronology? Buffon does not do so, whereas Darwin does.

No = 0; Yes = 1

#### Character 17: Tips of branches ending in present as well as in the past

There are several kinds of leaves, the ones bearing extant objects, the others carrying fossils. There are tips ending in present and tips ending in past times: either fossil or still-living species or groups can be assigned on leaves (0). This is the case with Pallas (0) but not with Darwin (1), for example. Alternatively, tips are restricted to objects of the present time (1). This character is coded “not applicable” when the tree doesn't carry time.

Yes = 0; No = 1

#### Character 18: Expression of an evolutionary destiny

Are the objects set on the leaves merely the product of fortuity (0) or has it been shepherded by any kind of destiny in evolution (1)? Chambers thinks so (1) whereas Darwin does not (0).

No = 0; Yes = 1

#### Character 19: Leaves are the only detectable objects

Some authors set concrete objects only at leaves. Then, there is no temporal distinction between leaves that can bear fossils and leaves that can bear extant entities. Some authors pretend to assign concrete objects somewhere else than leaves – at nodes – under the form of transitional forms, ancestors or missing links, seen as concrete objects. In such cases, leaves are not the only place for concrete objects. Mayr adheres to this last idea (1), whereas Hennig disagrees with it (0).

Yes = 0; No = 1

#### Character 20: Vertical cuts enhance differences between groups, whatever kind they are (1); between kingdoms of life (2)

A “kingdom” isn't seen as a mere group because it carries a cultural burden that dates back before elaboration of taxonomical rules. As a result, it has nothing to do with phylogeny and there are presumptions of polyphyly concerning kingdoms elaborated until the XIX^th^ century.

No vertical split: 0; Vertical splits between groups = 1; between kingdoms = 2. As an example, there is no vertical split for Darwin, splits between groups in Haeckel's *Anthropogenie* and between kingdoms for his *Monophyletischer Stammbaum der Organismen*.

#### Character 21: Blobs reflecting amounts of beings by generation

The more there are entities into the group, the broader the “blob” is. This is the case with Agassiz, for example.

Yes = 0; No = 1

#### Character 22: Blobs carry an evolutionary gradation (or else just used to draw a group)

In addition to the mere numerical information, is there an idea of “steps” which overcome the sequence of blobs? Agassiz does so, whereas Hitchcock does not. This character is coded “not applicable” when there is no blobs.

Yes = 0; No = 1

#### Character 23: Ancestor as an ancestral species or as past state of the same group

An “ancestral species” carries a concept of mutation, of fundamental difference between a mother and a daughter species. A “past state” insists on the continuity of a group or a family. Its name remains the same whereas the family's content has evolved. Buffon sees the ancestor as an ancestral species, whereas Lamarck sees it as the past state of the same group.

Ancestral species = 0; Past state of the group = 1

#### Character 24: Ancestors organized into succession of types in a genealogical way: 0; ancestors seen as sets of plesiomorphies: 1

“Ancestors” can bear several interpretations on the tree. They can be concrete individuals organized in a genealogical way that will set what are plesiomorphies (they express primitive steps or states). Alternatively, plesiomorphies refer to an hypothetical ancestor (which is expressed by them.) Mayr considers that ancestors are organized into successions of types in a genealogical way whereas Hennig considers ancestors as sets of plesiomorphies.

Concrete ancestors express plesiomorphies = 0; Plesiomorphies express virtual ancestors = 1.

#### Character 25: Primitiveness of lesser value

Do primitiveness, ancestral states or plesiomorphies include an idea of lesser value and/or imperfect state of adaptation? As an example, Mayr thinks that primitiveness do so whereas Hennig does not think that plesiomorphies do so.

Yes = 0, No = 1

#### Character 26: Taxonomic group with upper rank

Is the ancestor the taxonomic group itself? For example, is the ancestor of the group “Mammalia” the “Mammalia” group itself? Haeckel thinks so whereas Mayr does not.

No = 0; Yes = 1

#### Character 27: Diversification axis vertical

Diversification axis is vertical in Wallace's Attempts at a natural arrangement of birds, but is not vertical for Lamarck.

No = 0; Yes = 1.

#### Character 28: Hierarchical axis vertical: from the bottom to the top

Hierarchical axis is vertical from the bottom to the top in Darwin (1859)'s tree, but is not vertical for Buffon.

No = 0; Yes = 1.

#### Character 29: Hierarchical axis vertical: from the top to the bottom

Hierarchical axis is vertical from the top to the bottom for Duchesne, but is not such vertical for Buffon.

No = 0; Yes = 1.

#### Character 30: Hierarchical axis horizontal: from a side to the other

Hierarchical axis is horizontal in Wallace's Attempts at a natural arrangement of birds, but is not horizontal for Buffon.

No = 0; Yes = 1.

#### Character 31: Diversification axis horizontal: from the center to the outside

As an example, the diversification axis is horizontal from the top to the bottom for Chambers, but is not such for Buffon.

Yes = 0; No = 1.

#### Character 32: Diversification axis carries properties

Properties are attributes given to the objects in the tree. Those properties form a succession in the axis where they take place. Their content may be unspecified, as well as a sum of mutations. Duchesne sets properties on the diversification axis of his tree, whereas Darwin does not.

Yes = 0; No = 1

#### Character 33: Diversification axis carries value gradation of a value system

Are there degrees of perfection or complexity associated to this axis? Augier thinks so whereas Darwin does not.

No = 0; Yes = 1

#### Character 34: Diversification axis carries nested groups

Is there a hierarchy of groups implied by this axis? Duchesne adheres to this idea, whereas Chambers disagrees with it.

No = 0; Yes = 1

#### Character 35: Diversification axis carries time

Is time taken into consideration on this axis? Darwin does not do so, whereas Haeckel does in his *Anthropogenie*.

No = 0; Yes = 1

#### Character 36: Hierarchical axis carries series of species

Augier does so, whereas Hennig does not.

No = 0; Yes = 1

#### Character 37: Hierarchical axis carries properties

Sokal & Sneath consider properties on this axis, whereas Darwin does not.

Yes = 0; No = 1

#### Character 38: Hierarchical axis carries time

Sokal & Sneath do not consider time on this axis, while Darwin does.

No = 0; Yes = 1

#### Character 39: Hierarchical axis carries value gradation of a value system

Lamarck expresses such gradations, whereas Darwin does not.

No = 0; Yes = 1

#### Character 40: Generations are specified along the hierarchical axis

If yes, generations must be explicitly specified on the tree or in its description. Darwin (1859) does so, whereas Sokal and Sneath do not.

No = 0; Yes = 1

#### Character 41: Discontinuities well-marked horizontally

Such horizontal discontinuities are present for Zaluziansky, but absent for Buffon.

No = 0; Yes = 1

#### Character 42: Discontinuities well-marked vertically

Yes = 0; No = 1

Remark: this character is not redundant with the previous one, as there are trees with horizontal discontinuities but no vertical ones (such as Darwin's one in his “*Origin of Species”*). On the opposite, Adam Zaluziansky draws trees with vertical discontinuities but no horizontal ones. The tree realized by Agassiz shown no continuity at all between groups (neither horizontal nor vertical discontinuity), whereas Buffon's *“Table des chiens”* presents vertical and horizontal continuities (see Table 1).

**Table 1:** Distribution of some major authors according to horizontal and vertical continuities and/or discontinuities of their trees.

	No vertical discontinuities	Vertical discontinuities
No horizontal discontinuities	Buffon, *“Table des Chiens et de leurs variétés*	Darwin, *On the Origin of Species*
Horizontal discontinuities	Zaluziansky, *Methodi Herbariae Libri Tres*	Agassiz, *Recherches sur les poissons fossiles*

#### Character 43: The tree has a classificatory aim

The trees elaborated by Hennig (1966) have a classificatory aim, whereas the ones made by Buffon (1766) in his chapter about degeneration do not have any classificatory aim.

Yes = 0; No = 1

#### Character 44: Classification made before the tree

If yes, the tree merely illustrates a previously-made classification, what Mayr says, for example, in his “*Growth of biological thought*”. If no, classifying is made as the same time as the tree itself, such as what Hennig does.

No = 0; Yes = 1

#### Character 45: Tree's graph has an epistemological status (i.e. classifying concrete organisms, 0); Theoretical (i.e. model, 1)

Our trees can be either epistemological or theoretical. “Epistemological” trees are classifying concrete things: they depicts a hierarchy in shared attributes among known concrete entities (a dog, a cat, a mouse…), interpreted through a given model. Those trees authors, such as Mayr in his “*Methods and Principles of Systematic Zoology*”, want to represent a pattern of life much more than to explain the process of its diversification. On the opposite, a “theoretical” tree will not deal with concrete objects but with abstractions (A, B, C…) and aim at illustrating or supporting a theory about processes of diversification. It represents a model or the theoretical background itself: it makes conjectures about how should patterns be organized according to the known processes. This is, in particular, what Hennig does.

Epistemological = 0; Theoretical = 1

#### Character 46: Tree's graph has both status, one of them being much heavier

Finally, some authors aim to be simultaneously theoretical and epistemological. These authors will be coded 1. The most notable of the two components is coded in character 45. This is the case of Lamarck, who presents a theoretical and epistemological tree. Because his figure is more theoretical (the theory of increasing complexity) than epistemological (an idea of the sequence of concrete groups through generations), it is coded 1 for character 45 and 1 for character 46.

No = 0; Yes = 1

#### Character 47: The tree intends to illustrate the natural order

The tree illustrates a fundamentally ordered nature for Darwin, whereas it can't illustrate this order for Sokal & Sneath. For instance, for Darwin Nature is intrinsically ordered and we should have access to this order; for Sokal and Sneath Nature is intrinsically ordered however we do not have access to this order; and for Linné Nature is intrinsically ordered however this order has a supernatural origin and we have access to it.

No = 0; Yes, Nature is fundamentally ordered = 1.

#### Character 48: explicit tree

Is the tree explicit, i.e. drew or described, or is it merely evoked through “reading between lines”? The tree is implicit for Goethe, whereas it is explicit for others, for example, clearly described by Buffon or drew by Darwin.

Yes, drew or written = 0; No, implicit = 1

#### Character 49: the tree is genealogical

As an example, the tree is genealogical for Darwin, but it is not for Sokal & Sneath.

No = 0; Yes = 1

#### Character 50: If yes: superimposed meanings

If the tree is “not only genealogical”, external considerations are added to the mere genealogy (as value gradation, degrees of development, etc). This is what Teilhard de Chardin and Mayr do.

Strictly genealogical = 0; Not only = 1

#### Character 51: Gradation in perfection

Beings are organized through a gradation in perfection. A gradation in terms of perfection is meant by Lamarck, for example.

No = 0; Yes = 1

#### Character 52: If yes: Affects interpretation of all characters

The gradation in terms of perfection affects the interpretation of all characters for Lamarck, but only some of them for Gaudry.

Affects interpretation of all characters = 0; Affects only some of them = 1. If there is no gradation of values at all, an “irrelevant” state will be coded.

#### Character 53: Teleology

Teleology goes further than a mere trend towards an increasing complexity. It aims to converge in a given direction to a given end. For example, Teilhard de Chardin or Haeckel are teleologists.

Yes = 0; No = 1

#### Character 54: Is teleology explicitly or implicit?

Teleology is “implicitly mentioned” if it is a logical conclusion of the author's classificatory theory. There is no supernatural mechanism that places mankind at the top of evolution. However, mankind is set at this location as a logical consequence of the description of evolution by the author. This is what does Haeckel in his “*Anthropogenie*” or Romer in his “*Vertebrate Paleontology*”.

Explicitly = 0; Implicitly = 1. If there is no teleology an “irrelevant” state will be coded.

#### Character 55: Fossils included in trees

Fossilized individuals have been observed since centuries. But the interpretation about what they are has much changed. They can be interpreted as geological curiosities, never having been living forms. Some other authors, like Buffon, never include them in their classifications. Are fossilized living forms included in trees or not?

No = 0; Yes = 1

#### Character 56: Fossils are undiscovered extant forms

Fossils can be considered not as extinct species but as species still alive but undiscovered yet. This is especially what Lamarck thinks.

No = 0; Yes = 1

#### Character 57: Trees take into consideration the extinctions of species

If yes, the tree must illustrate at least one extinction. An extinction is the complete disappearance of a bloodline, whatever its cause might be. Buffon considers such extinctions; whereas Lamarck does not do so in his tree.

No = 0; Yes = 1

#### Character 58: The ancestor is concrete

An ancestor is said to be “concrete” when an object such as a fossil, is considered as the ancestor of a group. This is especially what Gaudry and Romer do.

Yes = 0; No = 1

#### Character 59: The ancestor is concrete only under some conditions

If, theoretically, a concrete ancestor is expected, the author can set conditions before attributing the status of “ancestor” to a concrete being. Here, all nodes don't carry a named ancestor. As an example, this is what does Romer in his “*Vertebrate Paleontology*”.

No = 0; Yes = 1

#### Character 60: Consideration of time in the tree

Time is absent from the tree of Pallas or from Wallace's one in his “*Natural arrangement of birds*”, whereas it is present in Darwin's trees.

No = 0; Yes = 1

#### Character 61: Time is explicit

Is time explicitly set into the tree graph (such as geological periods, thousands of years, etc.), as Agassiz does for example, or is it implicit – as, for example, Darwin's “generations”?

Time is explicit = 0; Implicit = 1 (Coded as irrelevant if there is no time).

#### Character 62: Kinship between plants and animals

Is there a link in terms of bloodlines between the two main kingdoms of life? This kinship is real according to Haeckel in his “*Monophyletischer Stammbaum der Organismen*”, whereas Lamarck considers that there is no such kinship.

No = 0; Yes = 1

#### Character 63: Same method to study plants and animals

Some authors tolerate, although relationship between plants and animals is not stated, the fact that the same methods can be used to study plants and animals. Here, the comparison must be proven in the scheme or in its description, for example, as Augier does when he describes the elaboration of his tree.

No = 0; Yes = 1

#### Character 64: Interbreeding between groups above species level

Interbreeding between groups above the species level is generally impossible with few exceptions for Darwin or Lamarck. It is however regarded as generally possible by Buffon (1766) in the context of his theory of degeneration. Then, for some authors as Hitchcock, it is considered as an impossible phenomenon.

Generally no = 0; Generally yes = 1; Never = 2

#### Character 65: Extent of the tree

Some authors classify only a small part of life onto one tree. This is, for example, what Duchesne does with his tree of strawberry plants. But other authors, like Pallas, want to make a comprehensive tree of the links between all groups of living organisms.

Only a part of beings = 0; All beings = 1

#### Character 66: Use of common methods for separate trees

If the tree is worth only for parts of the beings, a same method can nevertheless be used for different parts of life. Typically, Hitchcock uses the same methodology for his two separate trees, one for plants and the other one for animals. The two trees are besides facing on the same page. The character is coded irrelevant if the tree is used for all beings; and if there is only one tree for a single group of life in the work, then it will be coded 1.

Yes = 0; No = 1

#### Character 67: Mankind at the top

Typically, Haeckel's tree (“Anthropogenie”) is the most common illustration of a tree placing mankind at its top. Conversely, it is absolutely not the case with that of Darwin.

No = 0; Yes = 1

#### Character 68: If mankind is not at the top, a special place is assigned to a group to which mankind belongs

However, mankind can be seen as too noble to be ranked alongside animals and even plants. Then, it will not be mentioned into the classification. However, there may be a special attribution given to a group to which mankind belongs (e.g. primates or mammals), to emphasize on their sets of properties, as for example Barbançois or Pallas do. Coded as irrelevant if Yes at character 67.

No = 0; Yes = 1.

#### Character 69: Groups are really in Nature

If groups are not in Nature, they are openly considered as virtual. Note that considering groups as really in Nature or not is not necessarily linked to a position concerning the character 47, i.e. if there is a fundamental natural order or not. For instance, for Darwin groups are virtual but Nature is fundamentally ordered by genealogy, while for Buffon groups are virtual and Nature is disordered (Table 2). In parallel, groups are really in a perfectly ordered Nature for Linnaeus (a consequence of his creationism, fixism and essentialism). Finally, groups can really be in Nature but disordered by several exceptions, as for Étienne Geoffroy Saint-Hilaire (Table 2). According to Geoffroy Saint-Hilaire in his “Principes de philosophie zoologique”, Nature is fundamentally disordered: “La nature ne se laisse imposer aucune règle arbitraire” (p. 8)

**Table 2:** Distribution of some major authors according to species realism and taxonomic groups realism.

	Groups are virtual & conventional (nominalism)	Groups are really in Nature (essentialism)
Nature is fundamentally ordered	Darwin, Lamarck	Linnaeus
Nature is fundamentally disordered	Buffon	Geoffroy Saint-Hilaire*

(*: ambiguities are managed by the fact that an organism can belong to several separate groups.)

“Nature refuses to be imposed of any arbitrary rule”

But most groups are really in Nature: “Chaque classe, non comprise celle des reptiles qui est artificiellement formée, voit pour elle revenir un nombre donné de matériaux, neuf, huit et sept: si cela n'est pas toujours à l'égard de quelques familles, l'exception vient confirmer la règle” (p. 175)

“Each class, excluding that of the reptiles that is artificially formed, sees the presence of a given number of materials, nine, eight and seven, if this is not always towards some families, the exception confirms the rule”.

Some species can exceptionally belong to several groups at the same time, such as Monotremes (“*Cours de l'histoire naturelle des mammifères”, 4^e^ leçon*, p. 11):

“[Les mammifères] enfantent leurs petits vivans; les oiseaux pondent des œufs. Nous trouvons dans ce fait les moyens d'établir une ligne de démarcation bien tranchée entre les deux classes d'animaux à cœur bi-loculaire; toutefois quand nous parlerons des monotrêmes et des marsupiaux, peut-être serons nous forcés de reconnaître que cette distinction n'est point établie sur des caractères aussi nets et aussi précis”

“[Mammals] give birth to their young alive, the birds lay eggs. We find in this fact the means to establish a strict line of demarcation between the two classes of animals at heart bi-celled; but when we speak of Monotremes and marsupials, perhaps will we have to recognize that this distinction is not based on so clear and accurate characters”.

Groups are virtual = 0; Groups represent a non-arbitrary order = 1

#### Character 70: Groups are not used a priori but created with a classificatory purpose and justified by properties

This is especially what Buffon does when grouping together the horse, the donkey and the zebra: the group is supported by properties (the whole of their similarities).

Yes = 0; No = 1

#### Character 71: Groups aimed at assigning a specimen

Groups are given *a-priori* and imply some properties. The purpose is to assign specimens to some of them according to the properties found. Typically, this is what Lamarck does within the framework of his theory of evolution.

No = 0; Yes = 1

#### Character 72: Groups made according to genealogical links joining entities

In an evolutionary consideration, there are genealogical links between entities. Do groups refer to this genealogy? It is the case for Darwin, for whom *“All true classification is genealogical”*.

No = 0; Yes = 1.

#### Character 73: Mode of ranking (how ranks are made)

Ranking can be performed according to global similarity or to kinship links. This is for instance one of the oppositions between Linnaeus and Buffon: according to the first one, ranks are made according to global similarity; whereas, for Buffon, ranks should be made according to genealogical links.

Ranking made according to global similarity = 0; To hierarchy from kinship links = 1.

#### Character 74: Geometry of classification

A branching graph must carry information on its terminal/lateral branches. If there is no such information, i.e. if there is no difference between leaves and the basement of branches, such as Romer's tree in his *“Major steps in vertebrate evolution”*, then the content of the trunk becomes mono-dimensional, and the scheme becomes assimilated to a ladder (*scala*) and used as such for classification.

Classification under the form of a ladder (*scala*) of beings = 0; Made from a tree = 1.

#### Character 75: Groups made according to shared characters

There are numerous authors creating groups according to shared properties (character 70), however these properties can be diverse. They can be shared characters or shared degree of perfection. For instance, if Hennig elaborates his groups only according to shared characters, Lamarck groups animals according to an idea of perfection degree.

Yes = 0; No, groups are made by perfection degree or adaptive level = 1

#### Character 76: Groups are independent the ones from the others

Agassiz, a catastrophist author, sees series of acts of supernatural creations and extinctions of species. Then, according to this idea, groups of species are independent the ones from the others; there in no natural link between them. On the opposite, for Darwin, species have to be grouped according to bloodlines and groups are nested within each other.

Yes = 0; No, groups are linked or nested within each-other = 1

#### Character 77: Groups have always existed

Is there an emergence and an extinction of groups? This fact may be explained with evolutionism as well as with catastrophism. According to Linnaeus, groups have always existed whereas, for Agassiz, they are circumscribed in time.

Yes = 0; No, groups are circumscribed in time = 1

#### Character 78: Groups are perfectly defined

Is there a clear definition about what a group is or what it contains? As an example, for Hennig, groups are perfectly defined as they must be monophyletic ones, whereas Etienne Geoffroy Saint-Hilaire defines groups as vague sets of properties.

Yes = 0; No = 1

#### Character 79: Ranks express a sharing of characters by taxa

Do classificatory ranks insist on what is common or on what differs between what they contain? Ranks can be assigned to groups in an agglomerative procedure (0), according to shared characters. Ranks can also be assigned according to a divisive procedure (1). This is for instance what is done when classifiers confuse identification keys and phylogenies. They insist more heavily on what distinguishes groups, degrees of divergence between entities to classify (taxa). In the agglomerative approach there will be many nested ranks (like in cladistics), while in the divisive approach the trend will be to multiply groups of equal rank (like in phenetics). For instance, the divisive logic by which the class of birds is justified by Mayr explains why birds and reptiles have both the rank of a class.

Ranks express a sharing of characters by taxa = 0; A degree of divergence between taxa = 1

#### Character 80: what do ranks mean: genealogy only

No = 0; Yes = Ranks mean only the sharing of a common ancestor = 1

#### Character 81: Reality given to categories

Do categories, as species, genera, families, classes… etc. refer to objective, clearly definable, real entities in Nature?

Yes = 0; No = 1

#### Character 82: If yes:

For all categories = 0; Only for some categories = 1

#### Character 83: Use of parsimony

No = 0; Yes = 1.

#### Character 84: The classification is only made on the basis of shared presence of properties…‥

… or it also includes lack of properties (as invertebrates, agnatha, etc), for instance what does Linnaeus with Cryptogames.

No, It also includes lack of properties = 0; yes = 1

#### Character 85: Characters are only made of observation of attributes…‥

… or they also include subjective judgments like degree of complexity, degree of perfection, explicit processes like “cerebralization” (as Teilhard de Chardin does).

No, characters are not only made of observation of attributes = 0; Yes = 1

#### Character 86: Classification (i.e. arrangement) by global similarity

Lamarck and Buffon classify life using global similarity among organisms, whereas Hennig considers each of their characters.

Yes = 0; No = 1.

#### Character 87: Classification strictly made from global similarity

There can be other considerations added to the one of classification by global similarity. For instance, Lamarck adds an idea of increasing complexity (1). If not, the classification will be said “strictly” by global similarity (0). This is for instance what Buffon does.

Yes = 0; No = 1.

#### Character 88: Monophyly

Monophyly is considered in its present meaning: all descendants from a common ancestor. So, Hennig uses monophyly whereas Romer does not.

No = 0; Yes = 1

#### Character 89: If yes, monophyly is used strictly

Is monophyly used strictly and lonely or are there any other considerations added on? It is used strictly by Darwin or Hennig (0), but other considerations are added on by Haeckel. There is a specific case with Mayr, especially in his late writings: Mayr accepts in a first step of his studies the idea of cladograms, and even “monophylies”. But, according to him, a phylogenetic study must not stop at this stage and must consider the idea of grades. So Haeckel and Mayr use monophyly not strictly (1).

Strictly = 0; Other considerations added = 1

#### Character 90: possibility to detect and identify homoplasies

Homoplasies are similar characters that have not been inherited from a exclusive common ancestor. Lamarck's methodology in unsuitable to detect homoplasies, whereas Hennig's one is.

No = 0; Yes = 1.

#### Character 91: Inheritance of acquired characters

The presence of similar characters in individuals can be seen as similar adaptations acquired during a being's life. Then, adaptive convergences are much enhanced by living in a same place. This kind of inheritance of characters is fundamental in Lamarck's theory, whereas it is absent from Mayr's writings.

Yes = 0; No = 1

### 7. Analysis

Standard parsimony approach was conducted using PAUP* 4.0b10 [12]. Characters were treated as unordered and unweighted in the search of most parsimonious trees. Heuristic searches were performed with 1000 random addition sequences and TBR branch swapping. The results are shown under a 50%-majority-rule consensus tree. Characters are optimized on that tree using the ACCTRAN option, favoring reversions over convergences. Trees are rooted on Linnaeus (1758) and Adam Zaluziansky (1592) because the first elaborated classifications without trees and the second created trees without neither classificatory nor explanatory purposes.

## Results

The matrix contains 41 taxa and 91 characters, all informative for parsimony (Table 3).

**Table 3:** Matrix for 41 written or drawn “trees” and 91 characters.

	0	0	0	0	0	0	0	0	0	1	1	1	1	1	1	1	1	1	1	2	2	2	2	2	2	2	2	2	2	3	3	3	3	3	3	3	3	3	3	4	4	4	4	4	4
	1	2	3	4	5	6	7	8	9	0	1	2	3	4	5	6	7	8	9	0	1	2	3	4	5	6	7	8	9	0	1	2	3	4	5	6	7	8	9	0	1	2	3	4	5
1592 : Zaluziansky, *Methodi Herbariae Libri Tres*	0	0	0	0	0	0	0	0	0	0	0	0	0	0	0	0	0	?	?	0	?	?	-	-	-	-	0	0	0	0	0	0	0	0	0	0	0	0	0	0	0	0	0	0	0
1758 : Linnaeus, Systema Naturae, 10^th^ edition	?	?	?	?	?	?	?	?	?	?	?	?	?	?	?	?	?	?	?	?	?	?	?	?	?	?	?	?	?	?	?	?	?	?	?	?	?	?	?	?	?	?	0	0	?
1755 : Buffon, *«Table des Chiens et de leurs variétés»*	0	0	0	0	1	0	1	1	1	0	1	1	0	1	0	0	1	0	?	0	?	?	0	0	?	0	1	0	0	0	0	1	0	0	0	1	1	0	0	0	0	1	0	0	0
1766 : Buffon, *«De la Dégénération»*,	0	0	0	0	1	0	1	1	1	0	1	1	0	1	1	0	1	0	?	?	?	?	0	?	?	?	?	?	?	?	?	?	?	?	0	?	?	?	?	?	0	1	1	-	1
1766 : Duchesne, *Histoire Naturelle des Fraisiers*	0	0	0	0	1	0	1	1	1	0	1	1	0	1	1	0	1	0	?	0	?	?	0	0	?	0	0	0	1	1	0	0	0	1	0	1	1	0	1	0	1	1	0	0	0
1766 : Pallas, *Elenchus Zoophytorum*	0	0	0	0	0	0	0	0	1	1	1	1	0	0	0	0	0	0	?	?	?	?	?	?	?	0	0	1	0	1	0	1	0	0	0	1	1	0	1	0	0	1	0	0	1
1770 : Buffon, *Histoire Naturelle des Oiseaux*, tome 16	0	0	0	0	?	?	?	1	1	0	?	?	?	?	?	?	?	?	?	?	?	?	?	?	?	?	?	?	?	?	?	?	?	?	?	?	?	?	?	?	?	?	0	1	1
1774 : Rühling, *Ordines Naturales Plantarum*	0	0	0	0	0	1	1	0	1	1	0	0	0	0	0	0	0	0	?	?	?	?	?	?	?	0	0	0	1	0	0	1	1	0	0	1	1	0	0	1	0	1	0	0	0
1790 : Goethe, *Versuch die Metamorphose der Pflanzen zu erklären*	1	1	0	?	0	0	0	0	0	0	?	?	?	?	?	?	?	?	?	?	?	?	?	?	?	?	?	?	?	?	?	?	?	?	0	?	?	?	?	?	?	?	1	-	?
1801 : Augier, *Essai d'une nouvelle classification des végétaux*	0	0	0	0	0	1	1	0	1	1	0	0	0	0	0	0	0	?	?	0	?	?	?	?	?	0	0	1	0	1	0	1	1	0	0	1	1	0	1	0	0	1	0	0	0
1809 : Lamarck, *Philosophie Zoologique*	0	0	0	0	0	1	1	0	1	1	0	0	0	1	1	0	1	1	?	0	?	?	1	0	0	0	0	0	1	0	0	1	0	0	0	0	1	1	1	0	0	1	0	1	1
1816 : Barbançois, *«Observations sur la filiation des animaux»*	0	0	0	0	0	1	1	1	1	1	0	0	0	1	0	1	?	1	1	0	?	?	0	0	0	0	0	0	1	1	0	1	1	0	0	0	1	1	1	0	1	1	0	1	0
1816 : Barbançois, *«Observations pour servir à une classification (…)»*	0	0	0	0	0	1	1	0	0	0	0	0	0	1	?	?	?	?	?	0	?	?	?	?	?	0	0	1	0	0	0	1	0	1	0	0	1	1	1	0	0	1	0	1	0
1843 : Agassiz, *Recherches sur les poissons fossiles*	0	0	0	1	0	1	1	0	0	0	0	1	1	0	0	1	1	?	?	0	0	0	1	0	0	0	0	1	0	0	0	1	0	0	0	0	1	1	0	0	1	0	0	1	0
1845 : Chambers, *Vestiges of Natural History of Creation*	0	1	0	0	0	1	1	1	1	1	0	0	0	0	0	1	1	1	1	0	?	?	1	0	0	0	0	1	0	1	1	1	1	0	0	0	0	1	1	0	1	1	0	1	1
1850 : Bronn, *«Recherches sur les lois d'évolution (…)»*	0	1	1	1	0	1	1	1	1	1	?	?	0	0	1	1	0	1	?	0	0	0	0	1	?	0	0	1	0	0	0	1	0	0	0	0	1	1	1	0	0	0	0	1	1
1853 : Hitchcock, *Elementary Geology with an Introductory Notice*, 8^th^ edition	1	0	1	?	?	?	?	1	1	0	0	0	0	1	0	1	1	?	?	0	0	1	1	0	?	?	0	1	0	1	0	1	0	0	0	0	1	1	?	0	1	1	0	1	0
1855 : Wallace, *On the Law which has Regulated (…)*	0	0	0	1	0	0	0	1	1	0	1	1	0	1	?	?	?	?	?	0	?	?	0	0	1	0	?	?	?	?	?	?	?	?	0	?	?	?	?	?	?	?	1	-	1
1856 : Wallace, *«Attempts at a Natural arrangement of Birds»*	0	0	0	0	0	0	0	0	0	0	0	0	0	0	0	1	1	?	?	0	?	?	?	?	?	0	1	1	1	1	0	1	0	0	0	1	1	0	0	0	0	1	0	0	0
1859 : Darwin, *On the Origin of Species*, 1^st^ edition	0	0	0	1	0	0	0	1	1	0	1	1	0	1	1	1	1	0	?	0	?	?	0	1	1	0	0	1	0	0	0	1	0	0	0	0	1	1	0	0	1	1	0	0	1
1866 : Gaudry, *Considérations générales sur les animaux fossiles (…)*	0	0	0	1	0	1	1	0	0	0	1	1	0	1	1	1	1	1	?	0	?	?	1	0	?	0	0	1	0	0	0	1	0	0	0	0	1	1	?	0	1	1	0	0	0
1866 : Haeckel, *«Monophyletischer Stammbaum der Organismen »*	0	0	0	0	0	1	1	1	1	0	0	0	0	1	0	0	1	?	?	2	?	?	?	?	?	1	0	1	0	0	0	1	0	0	0	1	1	0	1	?	1	1	0	0	-
1874 : Haeckel, *Anthropogenie*	0	0	0	0	0	1	1	1	1	0	0	0	0	1	0	?	?	?	?	1	?	?	0	1	0	1	0	1	0	1	0	1	1	1	1	1	1	0	?	?	0	1	0	0	1
1877 : Haeckel, *Natürliche Schöpfungsgeschichte*	0	0	0	1	0	1	1	1	1	0	0	1	1	1	0	1	1	?	?	1	?	?	0	0	0	1	0	1	0	1	0	1	0	0	0	0	1	1	?	?	1	1	0	1	1
1882 : Richenow, *Vögelder zoologischen Gärten*	0	0	0	1	0	0	0	0	0	0	0	0	0	0	0	1	1	?	?	0	0	?	?	?	?	1	0	1	0	1	0	1	0	0	0	1	1	0	?	?	0	1	0	0	0
1888 : Darwin, *On the Origin of Specie*s, 8^th^ edition	0	0	0	1	0	0	0	1	1	0	1	1	0	1	1	1	1	0	?	0	?	?	0	1	1	0	0	1	0	0	0	1	0	0	0	0	1	1	0	0	1	1	0	0	1
1940 : Cuénot, *«Un essai d'arbre généalogique du règne animal»*	0	0	0	0	0	1	1	1	1	0	0	0	0	1	0	1	1	?	?	0	0	0	0	0	0	1	0	1	0	1	0	1	0	0	0	0	1	1	1	0	1	0	0	0	1
1953 : Mayr, *Methods and Principles of Systematic Zoology*	0	0	0	1	0	0	0	0	0	0	1	1	0	1	0	1	1	?	?	0	0	0	0	1	?	0	0	1	0	0	0	1	0	0	0	0	1	1	?	?	1	1	0	0	0
1955 : Teilhard de Chardin, *Le Phénomène Humain*	0	0	0	0	0	1	1	1	1	0	0	0	0	1	0	1	1	?	?	0	?	?	1	0	0	0	0	1	0	1	0	1	0	0	0	0	1	1	1	0	1	1	0	1	1
1956 : Teilhard de Chardin, *Le Groupe Zoologique Humain*	0	0	0	1	0	1	1	1	1	0	0	0	0	1	0	0	1	1	?	0	?	?	1	0	0	0	0	1	0	1	0	1	0	0	0	0	1	1	1	0	0	1	1	-	1
1962 : Simpson, *Principles of animal taxonomy*	0	0	0	1	0	0	0	1	1	0	1	1	0	1	1	1	0	0	1	1	0	1	0	0	1	0	0	1	0	0	0	0	1	0	0	0	1	1	0	0	1	1	0	1	1
1963 : Sokal & Sneath, *Principles of Numerical Taxonomy*	1	1	0	-	0	0	0	0	0	0	0	1	1	0	1	0	0	0	1	0	0	1	0	1	1	0	0	1	0	0	0	0	0	0	0	0	0	0	0	0	1	1	0	1	1
1966 : Romer, *Vertebrate Paleontology*	0	0	0	1	0	1	1	1	1	0	0	0	0	1	0	1	1	?	?	1	0	0	0	?	0	?	0	1	0	1	0	1	0	0	0	0	1	1	?	?	1	1	0	1	0
1966 : Sokal, *«Numerical Taxonomy»*	0	1	0	1	0	0	0	0	0	0	1	0	1	0	1	1	0	0	1	0	?	?	0	1	1	0	0	0	0	0	0	0	0	0	0	1	1	0	0	0	0	0	0	0	0
1966 : Hennig, *Phylogenetic Systematics*	1	1	0	1	0	0	0	0	0	0	1	1	0	1	1	1	1	0	0	0	?	?	0	1	1	0	0	1	0	0	0	1	0	0	0	0	1	1	0	1	1	1	0	0	1
1967 : Romer, *«Major Steps in Vertebrate Evolution»*	?	?	?	?	?	?	?	?	?	?	1	1	0	1	0	0	1	1	?	0	?	?	0	0	0	0	0	1	0	0	0	1	0	0	0	0	1	1	1	0	1	0	1	-	0
1973 : Romer, *«l'origine des classes de vertébrés»*	0	0	0	1	0	0	0	0	0	0	0	0	0	1	1	1	1	1	?	1	0	0	0	0	0	?	?	?	?	?	?	1	0	0	0	0	1	1	?	?	0	1	0	0	0
1973 : Sokal & Sneath, *Numerical Taxonomy*	1	1	0	-	0	0	0	0	0	0	0	1	1	0	1	0	0	0	1	0	0	1	0	1	1	0	0	1	0	0	0	0	0	0	0	0	0	0	0	0	1	1	0	1	1
1982 : Mayr, *The Growth of biological thought*	0	0	0	1	0	1	1	1	1	1	?	0	0	1	?	1	0	0	1	0	?	?	0	0	0	0	?	?	?	?	?	?	?	0	?	?	?	?	?	?	1	1	0	1	1
1988 : Sober, *Reconstructing the Past*	1	1	0	1	0	0	0	0	0	0	1	1	0	1	1	0	1	0	0	0	?	?	0	1	1	0	0	1	0	0	0	1	0	0	0	0	1	1	0	0	1	1	0	0	1
1991 : Tassy, *L'arbre à remonter le temps*	1	1	0	1	0	0	0	0	0	0	1	1	0	1	1	1	1	0	0	0	?	?	0	1	1	0	0	1	0	0	0	1	0	0	0	0	1	1	0	0	1	1	0	0	1

	4	4	4	4	5	5	5	5	5	5	5	5	5	5	6	6	6	6	6	6	6	6	6	6	7	7	7	7	7	7	7	7	7	7	8	8	8	8	8	8	8	8	8	8	9	9
	6	7	8	9	0	1	2	3	4	5	6	7	8	9	0	1	2	3	4	5	6	7	8	9	0	1	2	3	4	5	6	7	8	9	0	1	2	3	4	5	6	7	8	9	0	1
1592 : Zaluziansky, *Methodi Herbariae Libri Tres*	0	0	0	0	-	0	-	?	?	0	-	0	-	-	0	-	0	0	?	0	0	0	0	0	0	0	0	0	-	0	0	-	-	0	0	-	-	0	0	0	0	0	-	-	?	?
1758 : Linnaeus, Systema Naturae, 10^th^ edition	?	0	1	0	-	0	-	?	?	1	0	0	?	?	0	-	0	0	0	?	?	0	0	0	0	0	0	0	?	?	0	0	?	0	?	0	0	0	0	0	0	0	-	-	?	?
1755 : Buffon, *«Table des Chiens et de leurs variétés»*	0	1	0	1	0	1	0	?	?	0	-	0	0	0	0	-	?	?	1	0	1	?	?	0	0	0	0	1	1	1	0	1	0	1	0	1	-	0	0	0	0	0	-	-	0	0
1766 : Buffon, *«De la Dégénération»*,	0	1	1	0	-	0	-	?	?	1	0	1	0	0	0	-	1	-	1	1	-	1	-	0	0	0	?	0	1	?	1	1	?	1	0	1	-	0	0	1	0	0	-	-	0	0
1766 : Duchesne, *Histoire Naturelle des Fraisiers*	0	1	0	1	0	1	0	1	-	0	-	0	0	0	0	-	0	1	1	0	1	0	0	0	0	0	0	1	1	1	1	1	0	1	0	1	-	0	0	0	0	0	1	0	0	0
1766 : Pallas, *Elenchus Zoophytorum*	1	0	0	0	-	1	0	1	-	0	-	0	-	-	0	-	1	-	?	1	-	0	1	?	0	0	?	0	1	1	1	0	1	0	0	?	?	0	0	?	0	0	-	-	?	?
1770 : Buffon, *Histoire Naturelle des Oiseaux*, tome 16	0	1	1	1	1	0	-	1	-	0	-	0	?	?	1	0	?	?	1	0	1	1	-	?	0	0	0	0	1	0	1	?	?	?	?	?	?	0	0	1	0	1	0	-	0	0
1774 : Rühling, *Ordines Naturales Plantarum*	0	1	0	0	-	1	0	1	-	0	-	0	1	-	0	-	0	0	0	0	1	?	?	1	0	0	0	0	1	1	1	0	?	?	?	0	1	0	0	0	0	0	0	-	?	?
1790 : Goethe, *Versuch die Metamorphose der Pflanzen zu erklären*	?	1	1	1	0	0	-	1	-	0	-	0	1	-	0	-	?	1	?	0	1	0	0	?	?	?	?	?	?	?	?	?	?	1	?	?	?	0	0	1	0	0	1	1	?	?
1801 : Augier, *Essai d'une nouvelle classification des végétaux*	1	0	0	0	-	1	0	?	?	?	?	0	?	?	0	-	0	1	?	0	1	?	?	?	0	0	0	0	1	1	1	0	1	0	0	1	-	0	1	1	0	0	-	-	?	?
1809 : Lamarck, *Philosophie Zoologique*	1	1	0	1	0	1	0	0	0	1	1	0	0	0	1	0	0	0	0	0	1	1	-	0	1	1	0	0	?	1	1	0	1	0	?	1	-	0	0	0	0	1	0	-	0	0
1816 : Barbançois, *«Observations sur la filiation des animaux»*	0	1	0	1	1	1	0	1	-	?	?	?	0	0	1	1	?	?	?	0	1	1	1	?	0	0	0	0	1	1	1	0	?	0	0	?	?	0	0	0	0	1	0	-	0	0
1816 : Barbançois, *«Observations pour servir à une classification (…)»*	0	1	0	0	-	1	0	0	0	0	-	0	0	0	1	0	?	?	?	0	1	1	-	?	1	1	0	0	0	1	1	0	1	0	0	?	?	0	0	0	0	1	0	-	0	0
1843 : Agassiz, *Recherches sur les poissons fossiles*	0	0	0	1	1	1	0	0	0	1	0	1	0	0	1	0	?	?	?	0	1	1	-	-	0	0	0	0	?	?	0	1	0	0	0	1	-	0	0	1	0	0	0	-	?	?
1845 : Chambers, *Vestiges of Natural History of Creation*	1	1	0	0	-	1	0	0	0	1	0	0	0	0	1	0	0	?	?	0	1	1	-	?	0	0	0	0	1	1	1	1	?	1	0	?	?	0	0	0	0	1	0	-	0	0
1850 : Bronn, *«Recherches sur les lois d'évolution (…)»*	0	1	0	0	-	1	0	0	0	1	0	1	1	-	1	0	0	1	?	1	1	1	-	1	0	0	0	0	1	1	1	1	0	1	0	?	?	0	0	0	0	1	0	-	0	0
1853 : Hitchcock, *Elementary Geology with an Introductory Notice*, 8^th^ edition	0	1	0	1	0	1	0	0	0	1	0	1	0	0	1	0	0	0	2	0	0	1	-	1	1	1	0	0	1	-	1	0	1	0	0	?	?	0	0	0	0	0	0	-	0	?
1855 : Wallace, *On the Law which has Regulated (…)*	0	1	0	1	0	0	1	1	-	1	0	1	0	1	1	0	0	0	0	1	-	1	-	1	0	0	0	1	1	0	1	1	?	1	1	?	?	0	0	1	1	-	1	0	1	0
1856 : Wallace, *«Attempts at a Natural arrangement of Birds»*	0	1	0	0	-	0	-	1	-	0	-	1	?	?	0	-	?	?	?	0	1	0	0	?	0	0	?	0	1	0	1	1	?	0	0	1	-	0	0	0	0	1	0	-	1	?
1859 : Darwin, *On the Origin of Species*, 1^st^ edition	0	1	0	1	0	0	-	1	-	1	0	1	0	1	1	0	1	-	0	1	-	0	0	1	1	1	1	1	1	0	1	1	0	1	1	1	-	0	?	1	1	-	1	0	1	?
1866 : Gaudry, *Considérations générales sur les animaux fossiles (…)*	0	1	0	1	0	1	1	0	0	1	0	1	0	0	1	0	0	1	2	0	0	?	?	?	0	0	0	1	1	0	1	?	0	?	0	1	-	0	0	1	0	0	1	1	1	0
1866 : Haeckel, *«Monophyletischer Stammbaum der Organismen »*	-	1	0	-	-	1	0	0	0	0	-	0	?	?	-	-	1	-	2	1	-	0	1	?	?	?	0	?	1	?	1	?	0	?	?	?	?	0	0	0	0	0	?	?	1	?
1874 : Haeckel, *Anthropogenie*	0	1	0	1	1	1	0	0	1	1	0	1	0	1	1	1	?	?	?	0	1	1	-	0	?	?	0	1	1	1	1	1	?	1	1	1	-	0	0	1	0	0	1	1	0	0
1877 : Haeckel, *Natürliche Schöpfungsgeschichte*	0	1	0	1	0	1	0	0	0	1	0	1	1	-	1	0	0	1	-	0	1	1	-	0	?	?	0	?	0	1	1	?	1	1	1	1	-	0	0	?	0	1	1	1	0	0
1882 : Richenow, *Vögelder zoologischen Gärten*	0	?	0	0	-	0	-	1	-	0	-	0	1	-	0	-	?	?	2	0	1	0	0	?	?	?	0	?	?	?	?	?	?	0	0	?	?	0	?	?	?	?	?	?	?	?
1888 : Darwin, *On the Origin of Specie*s, 8^th^ edition	0	1	0	1	0	0	-	1	-	1	0	1	0	1	1	0	1	-	0	1	-	0	0	1	1	1	1	1	1	0	1	1	0	1	1	1	-	0	1	1	1	-	1	0	1	0
1940 : Cuénot, *«Un essai d'arbre généalogique du règne animal»*	1	1	0	1	0	1	0	0	0	1	0	1	0	0	1	1	1	-	?	1	-	1	-	0	0	1	0	0	1	1	0	?	0	1	1	1	-	0	0	1	1	-	1	1	0	1
1953 : Mayr, *Methods and Principles of Systematic Zoology*	0	1	0	1	0	0	-	1	-	1	0	1	0	0	1	0	0	1	1	0	1	?	?	0	0	0	0	?	?	?	1	1	1	0	0	1	-	0	0	0	1	-	1	1	0	1
1955 : Teilhard de Chardin, *Le Phénomène Humain*	1	1	0	1	1	1	0	0	0	1	0	1	1	-	1	0	0	0	?	0	1	1	-	0	0	1	0	0	1	1	1	1	0	0	0	1	-	0	0	0	0	1	0	-	0	1
1956 : Teilhard de Chardin, *Le Groupe Zoologique Humain*	0	1	0	1	1	1	0	0	0	?	?	?	1	-	1	0	0	0	2	1	-	1	-	?	0	0	0	0	1	1	1	1	0	1	0	?	?	0	?	0	1	-	0	-	0	1
1962 : Simpson, *Principles of animal taxonomy*	0	1	0	1	1	1	1	1	-	1	0	1	?	?	1	0	1	-	0	0	1	1	-	1	0	?	0	0	1	0	1	1	0	1	1	1	-	0	0	1	0	1	0	-	0	1
1963 : Sokal & Sneath, *Principles of Numerical Taxonomy*	0	0	0	0	-	0	-	1	-	1	0	1	0	0	1	0	1	-	0	1	0	0	0	0	0	1	0	0	1	0	1	1	0	0	0	1	-	0	0	1	0	1	0	-	0	1
1966 : Romer, *Vertebrate Paleontology*	0	1	0	1	0	1	0	0	1	1	0	1	0	1	1	0	?	?	?	0	1	1	-	?	?	?	0	0	1	0	1	1	1	0	0	1	-	0	0	?	0	0	0	-	0	1
1966 : Sokal, *«Numerical Taxonomy»*	0	0	0	?	?	0	-	1	-	1	0	1	0	1	1	1	?	?	?	1	0	0	0	0	0	1	0	0	1	0	1	1	0	0	0	?	?	1	0	1	0	0	0	-	0	1
1966 : Hennig, *Phylogenetic Systematics*	0	1	0	1	0	0	-	1	-	1	0	1	1	-	1	0	1	-	2	1	1	0	0	1	0	1	1	1	1	0	1	1	0	0	1	0	0	0	1	1	1	-	1	0	1	1
1967 : Romer, *«Major Steps in Vertebrate Evolution»*	0	1	0	1	0	1	0	0	0	1	0	1	0	0	1	0	1	-	?	0	1	1	-	?	0	?	0	0	0	1	1	1	?	-	-	1	-	0	?	0	0	1	0	-	0	1
1973 : Romer, *«l'origine des classes de vertébrés»*	0	1	0	1	0	1	0	0	1	1	0	1	0	1	1	0	?	?	?	0	1	1	-	-	1	1	?	0	1	0	1	1	0	-	0	-	-	0	0	?	0	0	1	1	0	1
1973 : Sokal & Sneath, *Numerical Taxonomy*	0	0	0	0	-	0	-	1	-	1	0	1	0	0	1	0	1	-	0	1	0	0	0	1	0	1	1	0	1	0	1	1	0	0	0	1	-	1	0	1	0	1	0	-	0	1
1982 : Mayr, *The Growth of biological thought*	0	1	0	1	1	1	1	1	-	1	0	1	0	1	1	0	1	-	0	1	-	1	-	1	0	0	1	0	1	0	1	1	0	0	0	1	-	0	0	0	0	1	0	-	0	1
1988 : Sober, *Reconstructing the Past*	0	1	0	1	0	0	-	1	-	1	0	1	0	0	1	0	1	-	?	1	-	0	0	1	1	1	1	1	1	0	1	1	0	0	1	?	?	1	1	1	1	-	1	0	1	1
1991 : Tassy, *L'arbre à remonter le temps*	0	1	0	1	0	0	-	1	-	1	0	1	0	1	1	0	1	-	0	1	-	0	0	1	0	1	1	1	1	0	1	1	0	0	1	1	-	1	1	1	1	-	1	0	1	1

**Figure 2:**
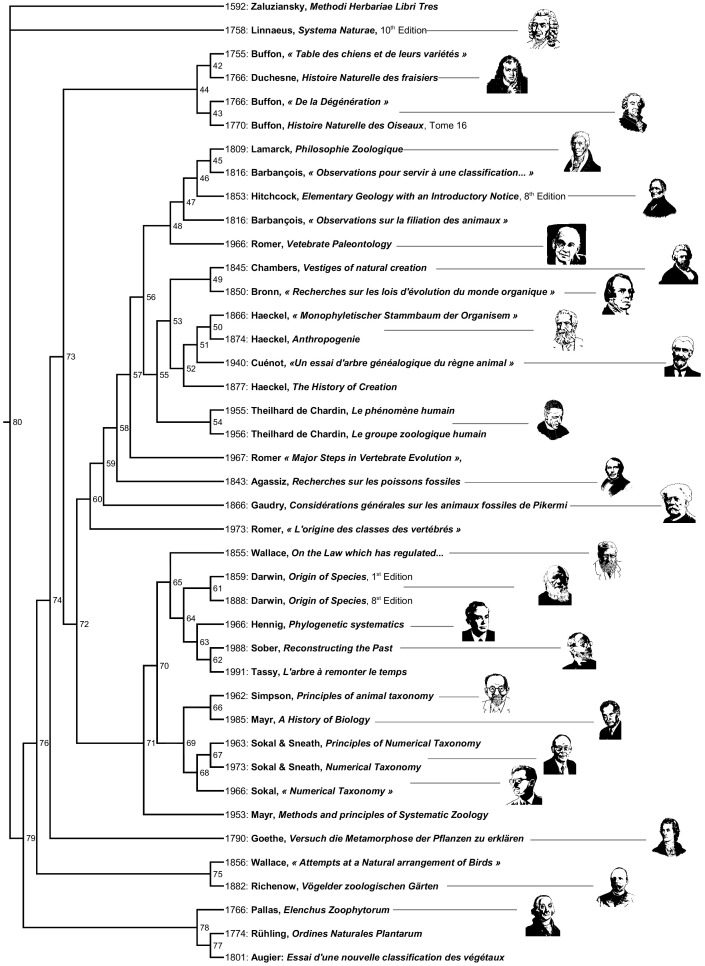
Majority-rule consensus tree of 279 trees of 378 steps. Nodes are numbered for convenience. Numbers in italics are percentages that refer to the proportion among equi-parsimonious trees.

The parsimony analysis provides 279 trees of 378 steps, with a C.I. of 0.24 and a R.I. of 0.61 (50% majority-rule consensus tree shown Fig. 2). In Fig. 2 it is possible to name some previously recognized groups.

### Node 78: Initial tree users

Initial tree users are non-evolutionist authors that are the first ones to use trees to depict life into the realm of Natural History.

The branches of their trees do not refer to purely logical links (char.9) but to gradations in value (char.10). This gradation is also carried by the hierarchical axis of the tree (char.39).

The tree graph is as theoretical as epistemological (char.46) and expresses a gradation in terms of values among beings (char.51).

A special place is assigned to a group to which mankind belongs (char.68). This last character has a C.I. of 0.5.

In terms of taxonomy, groups represent a non-arbitrary order (char.69) and are elaborated following a perfection degree or an increasing degree of complexity achieved through harmony between structures and their role in the environment (char.75). They are not perfectly circumscribed (char.78). Then, a reality is given not to all ranking categories, but at least for some of them (char.82). This character has a C.I. of 1 and strictly occurs in this group.

### Node 79: tree makers

Tree-makers are authors that use trees as tools for the classification of life.

The hierarchical axis of their trees is oriented vertically from the bottom to the top (char.28) or horizontally from a side to the other (char.30). None of the axes carries properties. (char.37). There are no more well-marked discontinuities (char.42).

To elaborate the tree, the authors consider common methods to study plants and animals (char.63). There is generally one tree in each work, and even if a kinship link between animals and plants is asserted, it is not translated into the use of similar methodologies for the study of the two (char.66).

Groups are no more independent but embedded into each other (char.76). Then, reality is given to phylogenetic categories (char.81).

### Node 72: evolutionists

Evolutionist authors consider not only a mere localized transformism, but a phenomenon of evolution in whole life, whatever its mechanisms are.

On the root of their trees is set an extinct object (char.4). The hierarchical axis does no more bear an idea of diversification (char.36), but a notion of time appears on it (char.38). Horizontally, discontinuities are well-marked (char.41).

With the idea of evolution comes new interpretations of fossils. Fossils are included into the tree graph (char.55). Trees take into consideration the extinctions of species (char.57). The notion of time is held into the graph (char.60). This character has a C.I. of 0.5.

Classificatory ranks express a sharing of characters by taxa, and no more a degree of divergence between them (char.79).

Finally, authors do not see an inheritance of acquired characters (char.91).

### Node 63: cladists

Cladists define themselves through the use of formally coded characters and formalized procedures to find phylogenies and the rejection of grades. The main principles have been defined in Hennig's Phylogenetic Systematics (1966).

For Cladists, the entity carried by the root is neither a concrete species nor ancestral group (char.1) but initial states of characters (char.2). The cladogram's lines do not express genealogical kinship links (char.8) but purely logical links (char.9).

Classificatory ranks express a sharing of characters between taxa (char. 79).

Finally, there is no inheritance of acquired characters (char. 91).

### Node 68: pheneticists

Created in 1963 by Sokal and Sneath, the phenetic school is mainly characterized by a renunciation to find the phylogeny of life. A new, mathematically based methodology is elaborated to use trees to represent degrees in global similarity. If, in node 69 (see below), including pheneticists, the classification is elaborated by global similarity (char.86), there is no redundancy with this character because of the separation made by some authors of this last group between classification and elaboration of the tree. Pheneticists group those two actions in a single one, the computation of the tree. And it is the methodology of that computation that is based on global similarity.

The entity carried on the root of the tree graph is a general states of characters (char.2) in the sense that the tree is rooted by an outgroup or on the most distant OTU to any other. The Phenogram's lines do not express genealogical kinship links (char.8) but purely logical links (char.9). The internal nodes express concepts (char.13) and no fossil is set on (char.14). Then, the hierarchical axis of the tree does not express time (char.38) (but degrees of global similarity).

With the renunciation to discover the phylogeny, the tree does not intend to illustrate the order of nature (char.47) and is not genealogical (char.49).

Phenetician authors use commons methods of study for separate trees (char.66). There is no special position assigned to mankind (char.67).

The groups elaborated are openly made as virtual (char.69) and aim at assigning a specimen (char.71).

Finally, there is a use of parsimony for phenetics (char.83). As an example, Sokal writes in his article: “A computer program developed by Camin and the author constructs cladograms with the fewest number of evolutionary steps” (p. 10).

## Discussion

### 1. New groups

Along with previously recognized groups of tree-thinkers, it is possible to point out in Fig. 2 some groups to which names can be given.

#### Node 44: “buffonians”

The two first identified “evolutionist” trees have been elaborated during the XVIII^th^ century. The first one has been written by Buffon and the other by his disciple, Duchesne. The “buffonians” school is a novel one. It is epistemologically characterized by a theory of transformism by “degeneration”.

The root carries an indication of value (char.7). Moreover, the entity carried on the root has a higher value (char.5): this character has a C.I. of 1 and is exclusive to buffonians. Branches of the tree express genealogical links (char.8) instead of purely logical links (char.9). Time is not taken into consideration into the tree (char.16) and the hierarchical axis is not set from the bottom to the top (char.28).

Finally, a strict monophyly (one ancestor and all its descendants) is used in buffonian trees (char.89).

Buffon considers two kinds of change along bloodlines, both used to create “monophyletic” groups. The first one is due to a reversible differentiation and the second one is the consequence of the phenomenon of degeneration. Reversible modifications are the consequence of changes in climate or in living conditions. They can be superimposed with one another, as for dogs, and they bring their current varieties. But when a dog returns to its “natural state”, he returns then to his primary characteristics. This type of change is represented by linking the initial state of a dog with its different biogeographical modifications.

“The Great Dane, the Mastiff and the Greyhound, although different at the first glance, are, however, the same dog: The Great Dane is no more than a Mastiff [with a hair] thicker, more enriched; the Mastiff a Greyhound slenderer, more tapering, and both neater; and there is no more difference between a Great Dane dog, a Mastiff and a a Greyhound, than between a Dutchman, a Frenchman and an Italian. Supposing therefore that the Mastiff is originated or rather natural to France, he will have produced the Great Dane in a colder climate, and the Greyhound in a warmer climate: and this is also verified by facts, as Great Danes comes us from the north, and Greyhounds come from Constantinople and the Levant” (Histoire Naturelle, Générale et Particulière, Tome 5, p. 205)

The phenomenon of degeneration, mutationist and non-reversible, is itself associated with a high requirement of monophyly. The successive degenerations of a species will be represented on the same tree than the parent one. This is what is described in Buffon's famous tree of horses, donkeys and zebras:

“From this point of view, the horse, the zebra and the donkey belong all three to the same family, if the horse is the strain/root or the main trunk, the zebra and the donkey will be the collateral stems/branches: because the number of their similarities (/the similarities between them) is infinitely greater than their differences, we can consider them as making only one genera, from which the main characters are clearly defined and common to all three: they are the only ones really solipeds, that means, who have the horn of their feet in a single piece without any appearance of fingers or nails; and although three species are distinct, they are however not absolute nor clearly separated, since the donkey product with the mare, the horse with the jenny, and it is probable that if one overcomes to domesticate zebra, and ease its wild and recalcitrant nature, it would also occur with the horse and the donkey, as they produce together”.(Histoire Naturelle, Générale et Particulière, Tome 14, pp. 335–336).

This consideration of monophyly is not isolated: there are numerous other examples in this chapter:

“Those animals who have antlers, although they are ruminants and shaped inside such as those who bear horns, seem to make a genera, a separated family, in which the moose is the main stem and the reindeer, the deer, the cheetal, the fallow deer and the roe are the minor and collateral branches; because there are only those six species of animals that have the head armed with a branching antler which falls and is renewed every year; and independently of this generic and common character, they resemble each other a lot in the conformation and natural habits: so we would rather obtain hybrids of the deer or of the fallow deer mixed with the reindeer and the cheetal than hybrids of the deer and the cow” (p. 349).

The monophyly exhibited in Buffon's groups is more than a mere intuition. The author sees the ability of interbreeding as a proof of this monophyly. He develops a protocol of which he discusses the results.

“The dog, the wolf, the fox, the jackal and the isatis form another genera, in which each is really so close to the others, and in which individuals are so much alike, especially in the internal conformation and in the parts of the generation, that is hard to understand why those animals do not produce together; it seemed to me by the experiences I made on the mix of the dog with the wolf and the fox, that the reluctance to coupling came from the wolf and the fox rather than from the dog, that is to say, from the wild animal rather than from the domestic animal; because the female dogs that I tested, would have willingly suffered the fox and the wolf” (p. 449).

This character has a C.I. of 0.5 and is shared with “connected graph users” (see below).

#### Node 60: “metaphoricians”

These authors directly draw *a priori* the “great tree of life” concerning concrete organisms without the analytical step: there is a fusion of the first level (empirical) and the third level (metaphorical and synthetic) in their use of the term “tree”. If the internal nodes of the trees do not refer to characters, races or species (char.11), the leaves express an evolutionary destiny (char.18). This character is exclusive to this group (C.I. 1).

The trees express a gradation in terms of perfection among beings (char.51). It is guided by theology, a direction given to evolution (char.53). This character has a C.I. of 0.5: they are not the only ones.

There is no possibility of interbreeding between two groups with an upper rank than the one of species (char.64). Finally, a special position into the tree is given to a group to which mankind belongs (char.68). This character has a C.I. of 0.5.

#### Node 70: “connected graphs users”

Connected graphs are mathematically based representations of trees. They are defined by the fact that it is possible, from any vertex, to join every other along the edges. “Connected graphs users” use highly topologically-based representations.

Branches of the trees express genealogical links (char.8) instead of purely logical links (char.9).“Blobs”, when used, do not involve a concept of evolutionary gradation, but are merely used to draw a group (char.22). When “blobs” are used, they are superposed to a primarily elaborated connex graph. Two phases are given to elaborate such trees. The first one is a connex graph that expresses mere genealogical links. The second phase consists in elaborating groups based on overall similarity and ecological adaptation. That is what Mayr describes in his *Growth of biological thought*, when he writes *“The cladist ignores the existence of grades because this approach condones the recognition of “paraphyletic” taxa. A monophyletic group is “paraphyletic,” in the terminology of the cladists, if it is not “holophyletic,” that is, if it does not include all of the descendants of the common ancestor. The class Reptilia, for instance, as traditionally recognized, is a paraphyletic group, because it does not include the Aves and the Mammalia, two groups that were separated as having reached a grade level differing from that of the remaining Reptilia. The recognition of paraphyletic groups prevents the automatic translation of a classification into a branching pattern but it is able to express degrees of divergence, something the cladogram cannot do”*. This character has a C.I. of 0.5.

The numerous considerations about the elaboration of trees and their representation of what a “phylogeny” is, peculiarly in the texts, make trees to have a theoretical status (char.45).

If the ancestor is seen as concrete, this is only under some conditions (char.59). There is a kinship between plants and animals (char.62). There is, generally, no ability to interbreed for different groups with an upper rank than the one of species (char.64). The tree worth for all beings (char.65).

Groups are not considered as virtual, but as representative of a non-arbitrary order (char.69).

Then, when monophyly is used, it is done strictly (char.89). This character has a C.I. of 0.5, shared with Buffonians (see above).

#### Node 65: Strictly genealogical classifications

This set is based on a common methodology.

Groups are elaborated according to kinship links (char.73) in the sense that a grouping must contain an ancestor and all its descent. Using modern terminology, groups are monophyletic. Classificatory ranks express a degree of divergence among taxa (char.79) and the sharing of a common ancestor (char.80).

Finally, homoplasies are detected (char.90) despite to an inheritance of acquired characters (char.91). The distinction between what is innate and what is acquired is late in the XIX^th^ century. But, for monophyletists, the inheritance of acquired characters is an inheritance of accidents, ant not an effort of organisms to adapt themselves to their environment. The inheritance of acquired characters according to Buffon or Lamarck is narrowly linked to an answer of the organism to its environment: climate, food, and even bad treatments due to mankind and domestication. This last phenomenon is seen as reversible, especially for Buffon. On the opposite, the inheritance of acquired characters for monophyletists have the same randomness than the inheritance of innate ones. Darwin ties an explanation since the first edition of *The Origin of Species* (p. 131): “*But the much greater variability, as well as the greater frequency of monstrosities, under domestication or cultivation, than under nature, leads me to believe that deviations of structure are in some way due to the nature of the conditions of life, to which the parents and their more remote ancestors have been exposed during several generations*”. Finally, the confusion between the two and the links between them is due to an ignorance of the mechanisms of variation, what Darwin confesses (p. 167 1^st^ edition of the *Origin of Species*): *“Our ignorance of the laws of variation is profound. Not in one case out of a hundred can we pretend to assign any reason why this or that part differs, more or less, from the same part in the parents”*.

No surprise to find in that group of “monophyletists” Charles Darwin in his first edition of the *Origin of species* (1859) where, as Nelson [13] reminded us, we find in chapter XIII a clear recommendation about monophyly that was removed in later editions because examples were taken from human races:

“*In tumbler pigeons, though some sub-varieties differ from the others in the important character of having a longer beak, yet all are kept together from having the common habit of tumbling; but the short-faced beak has nearly or quite lost this habit; nevertheless without any reasoning of thinking on the subject, these tumblers are kept in the same group, because allied in blood and alike in some other respects. If it could be proved that the Hottentots had descended from the Negro, I think he would be classed under the Negro group, however much he might differ in color and other important characters from Negroes*”.

This passage shows that Darwin recommended to classify organisms according to strict common ancestry whatever the divergence accumulated since common ancestors. Lecointre [14] replaced “Hottentots” by “Chicken” and “Negro” by “Reptiles”: “If it could be proved that the Chicken had descended from the Reptile, I think he would be classed under the Reptile group, however much he might differ in color and other important characters from Reptiles”. Darwin advocated for priority given to monophyly, implicitly dismissing what will be later called “paraphyletic groups” or “grades”.

Wallace centers the nodes of his trees on the concept of “antitype”, or ancestor. He links the formation of groups to the divergence of two lines from a node. In the 5^th^ page of his *On the Law which has Regulated the Introduction of New Species, Wallace* precises that: *“There constantly occur two or more modifications of an organ or modifications of two distinct organs, leading us on to two distinct series of species, which at length differ so much from each other as to form distinct genera or families. These are the parallel series or representative groups of naturalists, and they often occur in different countries, or are found fossil in different formations. They are said to have an analogy to each other when they are so far removed from their common antitype as to differ in many important points of structure, while they still preserve a family resemblance. We thus see how difficult it is to determine in every case whether a given relation is an analogy or an affinity”.*

Thus, the leaves are considered as the only detectable objects (char.19): this character is exclusive of the group (CI of 1).

#### Node 69: “similarity classifiers”

“Similarity classifiers” are connected graph users, but use a methodology based on overall similarity.

Tips of the tree's branches end in present as well as in past (char.17). The diversification axis explains properties (char.32).

The classification of entities is made before the elaboration of the tree (char.44), and this one is not strictly genealogical (char.50).

The classification is elaborated by global similarity (char.86) whatever the way the tree is made (i.e. by global similarity or not) and there is no use of monophyly (char.88).

#### Node 66: “Grade theoreticians”

Gradists are diphyletic. If “Gradism” appears as being fragmented, we can distinguish “grade users”, that are a paraphyletic part of “metaphoricians”, and “grade theoreticians” at node 66. Those last are characterized by common thoughts on classifications and epistemological argumentation for the merits of the use of grades.

In grade theoreticians, ancestors are organized into succession of types in a genealogical way into the tree (char.24). Moreover, the diversification axis of the tree implicitly expresses an idea of gradation of values (char.33).

Then, there is a gradation in terms of perfection between beings into the tree graph (char.51).

### 2. A low C.I. but clades consistent with traditionally recognized schools

The Consistency index is very low (0.24). Does it mean that the tree is meaningless? Note that the majority-rule consensus tree recovers a number of groups traditionally recognized like cladists, pheneticists, evolutionists, Buffon's recognized originality [15], [16]. The low C.I. means that the modalities of ideas transfers are diverse, or else the number of reversals is high, which is expected by the fact that an author rarely takes all from a predecessor. Indeed authors read previous authors (for instance Haeckel read Goethe, Darwin, Buffon, Cuvier, Lamarck; Darwin read Chambers and Lamarck; Barbançois read Lamarck), but never became full replicates. This could be true if we had chosen minor authors who rather behave as followers or even disciples – this could increase the C.I. But we only selected innovators, among which ideas about trees circulated in a rather mosaic fashion. Haeckel's tree of *Anthropogenie* (1877) has nothing to do with Darwin's tree though Haeckel claimed to be Darwinian [17]. Indeed over 378 steps, more than one third (134) are reversals under ACCTRAN optimization. This leaves 110 changes for convergences or exclusive changes. Successive readings is a phenomenon of transfer that partly explains why basal positions are occupied by authors on the eighteenth century, the clade 71 is mainly occupied by authors of the twentieth century and the clade 60 mainly occupied by authors of the nineteenth century. Another possible cause for sharing ideas is convergence: Wallace's ideas about trees were formulated in 1855 independently from Darwin's. However this seems to be exceptional.

### 3. Legitimizing the approach

There are different meanings given to trees in the field of Biology [18]. “Tree” is a term commonly used in Biology at three levels that are not always distinguished (e.g [19]). These are

- the epistemological level,
- the theoretical level, and
- the metaphorical/synthetic level.

The epistemological level is exemplified by the tree we construct at the lab from a data matrix using, for instance, standard parsimony [20], [21]. The theoretical level sets and exhibits the kind of relationships that link the objects under scrutiny, given the known processes of change or exchange. One of the most famous theoretical tree is the one published by Charles Darwin in “The Origin of Species” in 1859, which shows what should be the graphical form of the genealogical relationships among species if the theory is true [22]. The theoretical tree is a conjecture [22] about the form to be given to interrelationships according to a certain process of change. It does not need to be expressed with real, empirical entities to be useful: after all, Darwin (1859) and Hennig (1950) used “A, B, C, D…” as terminals in such trees. The metaphorical level is mostly used when telling the history of life, and/or at a step of synthesis of knowledge (e.g. for scientific popularization). It is neither a theoretical tree (second level) because it refers to real objects, nor an empirical one (first level) because it is never the direct output of a parsimony or maximum likelihood program. It is always redrawn to synthesize or tell a story (see for instance [23], [24], [25], [26]).

Such a distinction of three levels is of importance here to understand why some tools elaborated at the first level can be used in a foreign theoretical context (second level), i.e. that is not the one by which those tools originated. As Tëmkin and Eldredge stated [27] when using standard parsimony to the diversity of musical instruments, “*application of methodologies originally formulated for biological questions has earned general acceptance in historical linguistics ans stemmatics* (…), *though the underlying theory had already been developed in these fields prior to the widespread implementation of cladistics in biology* (…)”. Trees are also used by Moretti [28] to compare writings of the literature, though confusing the first and second levels. Trees constructed through the parsimony criterion were first used by Kluge and Farris (1969) [20] from a method of character analysis defined by a botanist [29] called “groundplan divergence analysis”. Such an algorithm chooses the tree which branches maximize contiguity among identical character states. By doing so, it minimizes the number of character changes onto the branches (it is the most parsimonious tree), but it also maximizes consistency among characters and consistency of the explanations driven from them. It minimizes *ad hoc* hypotheses of character change required by the tree for the conflicting [30]. The algorithm and tools to implement it have been exported outside systematics in fields that previously ignored it and where it appeared to be fruitful, for instance in biochemistry [31], [32], [33], [34], in linguistics [35], [36], [37], in musicology [27], and even to perform an ironical “cladistics of cladists” [38], [39].

Why should we choose the tree that maximizes contiguity of identical character states (i.e. the most parsimonious tree) and not another one? Here we reformulate the second question set above: Why should we choose the tree maximizing consistency among characters? Two answers can be given.

The first one refers to the theoretical (second) level: it is grounded by the theory of Biology. In comparative Biology, since Darwin (and even in some predarwinian transformists, [40], any character similarity between individuals that do not interbreed today must be taken as the product of common ancestry: the common character state must come from the times when common ancestors did interbreed. Present descendants have inherited from them the present character states. This theoretical-genealogical point of view can be viewed as the reason why we prefer to join branches with identical character states, i.e. to maximize common ancestry of equal character states rather than choosing another tree.

The second answer is, by far, more general, and is theory-free. Consistency is not only a property of our trees, it is a property of any rational enquiry. It is a conceptual indicator of truth in science in general [41]. As such, it is one of the first expected properties of any theory or scenario proposed through scientific means. Maximizing consistency among characters is just offering a rational interpretation of the character distribution across the compared entities, by using a hierarchy from the most general to the most particular. We prefer this hierarchical representation over networks in a first step because it is what we need to test for consistency of previous categories, propose new ones and exhibit sharings (even homoplastic ones if needed).

Comparing the ideas that authors wrote in their books is not biology indeed. According to the above second answer, using trees to compare ideas can be performed without the need to refer to the theoretical foundations of the use of trees in Biology. What we expect by drawing a tree of “ideas about trees” is to maximize the consistency of the distribution of ideas they contain, whatever the processes invoked in the specific theoretical realm of history of Science. It is therefore meaningful to use “trees” to compare any set of entities that exhibit similarities, at least at the heuristic level, a “tree” being viewed just as a figure that provides the rational hierarchical interpretation of the character state distribution. After all, the botanist Augustin Augier (1801) provides compelling evidence: he used such a “tree” that way –obviously in a non-computerized manner- in a non-transformist theory of life. And outside science, trees had been used for long ago to organize the world in a hierarchical manner without any evolutionary connotations [9].

### 4. Limitations of the approach

Character independence is often a desired property of the matrix prior to performing a phylogenetic analysis. However character independence is rarely neither controlled nor measured. In comparative anatomy, we often find in a matrix several characters from the skull without knowing anything about the degree of impact that would have the change on one of them on the others. This is the same here: does a given idea necessarily imply the presence of another? It is possible, however the high mosaicism of ideas and the rather low C.I. tend to show the contrary.

What is the benefit of this approach for history of science? For systematics? The benefit is into formalization allowed a comparative approach to ideas, and character coding. Some historians wish to understand an author “from within”, practising what we call in literature the “close reading” [28], sometimes even refusing to compare an author with another of a different period. Other historians compare authors among them because they are primarily interested in the history of ideas. They practice something similar to the “distant reading” in literature [28], a necessary step before comparison. They even categorize schools of thinking. This was made for “schools” of systematics (“pheneticists”, “synthetists”, “cladists”, “gradists”, etc.) without any possibility to formally control the consistency (should we say “monophyly?”) of these categories. For those ones, we propose a formalized way to expose similarities (through character coding) and control homogeneity and consistency of categories (the most parsimonious tree provides a test of monophyly and consistency index). We offer the possibility to create new categories.

### 5. Is that tree really a phylogenetic tree?

At the (second) theoretical level, is there some interest to accept common ancestry of scientific ideas, at least provisionally? *A posteriori*, does it make sense? Some authors [27] consider that it does. According to them, Darwin's principle of “descent with modification” to explain similarities “*is not restricted to the biological world and in a broad sense applies to any historical process that rests upon transmission of information from one generation to the next*”. From a heuristic point of view, the assumption that *at least some ideas* must have been subject to “descent with modification” makes sense in the realm of History of Science. Other ideas should have followed different paths, exhibiting patterns reflecting some departure from that basic assumption, just as in Tëmkin and Eldredge [27]: “*critical analyzes of the diversity patterns of two musical instruments, the stringed psaltery and the brasswind cornet, reveal paths of information transfer and the origins of innovation unique to the cultural context that are unlike those in biological systems*”. In other words, let's use descent with modification as a kind of null hypothesis, which will provide the tools to measure vertical inheritance *versus* convergent occurrences or reversals, but also effect of “horizontal diffusion” of ideas. Let's focus on that point.

Indeed the flow of ideas through times doesn't behave like in biological entities. Ascending source of information pointing backwards to a single ancestor is not the sole possible process: an author is most of the time influenced not by a single precursor but by multiple sources. The flow of information is permanently multiple in its roots and in its outcomes. If there is a process of descent with modification, it does not have the form of Darwin's tree, rather a form closer to a network, e.g., what we find in bacteria due to horizontal gene transfer. The fact that epochs of those ideas about trees are rather grouped instead of completely shuffled suggest that authors first of all share ideas of their century. This brings into question the need of using networks [42] instead of trees to represent how ideas are shared. But the question is useless in the present case. Let's explain why. When we study interrelationships of living organisms, there is a difference between trees and networks at the (first) empirical level because biological inheritance is embedded within a time framework due to the physical process of generation. This is drawn in Darwin's theoretical tree (second level). We mostly use networks when there is another process of character transmission that violates this generation time's rule (and sometimes also for representing effects of homoplasy without reference to any particular process). Typically, horizontal gene transfers are called “horizontal” because they violate the typical “vertical”, classical generation-driven inheritance. However, we don't need networks for ideas to depict departures from a major mode of transmission, because there is none: transmission of ideas can jump large periods of time. Mayr could read Aristotle [10]. Transmission of ideas is not constrained by a physical process that imposes its temporality. If two authors are sister-groups, this could perfectly be because an author has learnt directly from his master, or one picked up the idea from one of his contemporary colleague, or because one of both has read writings of the other dating back to centuries. This makes no difference: the fact is that one has read someone else's ideas and adopted them. The distinction between adopting someone else's idea (synapomorphy) and having the same idea twice by convergence (homoplasy) is maintained here. The fact that networks are not useful at the present step of the study does not mean that the cladogram is not useful. The cladogram is used here to maximize consistency among sharings of ideas about trees, whatever the ways employed in ideas circulation. It functions as a test for common origin (it also has potentially the power of revealing convergences), and provides test for categories.

If the transmission of ideas was really constrained through times, with a generation of authors linearly transmitting their ideas to their intellectual offspring, we would have obtained a comb with the earliest author as the most basal branch and the branches at the top being the most recent authors. It cannot be the reverse because an author can read his predecessors but cannot read future authors. The situation here is obviously more complicated: readings have been anachronistic in the sense that a given author could read any author of the past, whatever its ancientness.
